# Bike Score®: Associations between urban bikeability and cycling behavior in 24 cities

**DOI:** 10.1186/s12966-016-0339-0

**Published:** 2016-02-11

**Authors:** Meghan Winters, Kay Teschke, Michael Brauer, Daniel Fuller

**Affiliations:** Faculty of Health Sciences, Simon Fraser University, Blusson Hall Rm 11522, 8888 University Drive, Burnaby, BC V5A 1S6 Canada; School of Population and Public Health, University of British Columbia, 2206 East Mall, Vancouver, BC V6T 1Z3 Canada; Department of Community Health and Epidemiology, University of Saskatchewan, Health Science Building, 107 Wiggins Road, Saskatoon, Saskatchewan S7N 5E5 Canada

**Keywords:** Active transport, Cycling, Built environment, Multi-level modeling, Bike Score

## Abstract

**Background:**

There is growing interest in designing cities that support not only walking, but also cycling. Bike Score® is a metric capturing environmental characteristics associated with cycling that is now available for over 160 US and Canadian cities. Our aim was to determine if Bike Score was associated with between and within-city variability in cycling behavior.

**Methods:**

We used linear regression to model associations between Bike Score and journey to work cycling mode share (US: American Community Survey, 2013 or 2012 5-year estimates; Canada: 2011 National Household Survey) for 5664 census tracts in 24 US and Canadian cities.

**Results:**

At the city level, the correlation between mean Bike Score and mean journey to work cycling mode share was moderate (*r* = 0.52). At the census tract level, the correlation was 0.35; a ten-unit increase in Bike Score was associated with a 0.5 % (95 % CI: 0.5 to 0.6) increase in the proportion of population cycling to work, a meaningful difference given the low modal shares (mean = 1.9 %) in many North American cities. Census tracts with the highest Bike Scores (>90 to 100) had mode shares 4.0 % higher (*β* = 4.0, 95 % CI: 2.9 to 5.0) than the lowest Bike Score areas (0–25). City specific analyses indicated between-city variability in associations, with regression estimates between Bike Score and mode share ranging from 0.2 to 3.5 %.

**Conclusions:**

The Bike Score metric was associated bicycle mode share between and within cities, suggesting its utility for planning bicycle infrastructure.

**Electronic supplementary material:**

The online version of this article (doi:10.1186/s12966-016-0339-0) contains supplementary material, which is available to authorized users.

## Background

Considering both risks and benefits, active travel carries a net benefit on all-cause mortality [1–3]. In ecological studies, areas with higher levels of active travel are associated with lower traffic fatality risk, [4] higher levels of physical activity, [5] and lower rates of obesity and diabetes [5]. Many studies have documented links between neighborhood design and active transportation [6, 7]. In early research project-specific measures of walkability were developed, limiting comparability between studies [8, 9]. More recently, researchers have used Walk Score® (www.walkscore.com), [10–13] a web-based tool that scores neighborhood walkability based on proximity to various destinations. The popularity of the Walk Score metric is likely due in part to its extensive coverage of North America, providing consistent methodology across settings at relatively low cost. Walk Score is correlated with other measures of access to destinations and walkability [14], and with walking for transportation [11, 15, 16] at a level comparable with other walkability measures [15].

In contrast with walking, cycling has received less attention in the neighborhood design literature. Cycling is currently underused as a transportation mode in North America, although there may be increased uptake with improvements in infrastructure amid health, environmental, and mobility considerations [17, 18]. There are similarities in features that constitute a walkable or a bikeable neighborhood, however, certain environmental characteristics such as cycling-specific infrastructure and topography are additional factors relevant for cycling [19–22]. In order to promote a shift to active transportation for trips of moderate distance, beyond distances suitable to walking, metrics specific to cycling are useful for guiding neighborhood design.

In 2012 we partnered with Walk Score to incorporate findings from our empirical research on cycling and urban form, [19] which led to their development of “Bike Score” in North American cities. Bike Score is based on environmental characteristics consistently associated with cycling: density and quality of cycling infrastructure, topography, desirable amenities and road connectivity. As of 2015 Bike Score was available for over 160 US and Canadian cities. City- and neighborhood- rankings have been publicized by Walk Score, [23] but its correlation with cycling behavior has not been explored. We assess the extent which Bike Score predicts cycling behavior, both between and within cities, through an analysis of 5664 census tracts across 24 Canadian and US cities.

## Methods

In this ecological analysis, cities and census tracts are the units of analysis. Census tracts in both the US and Canada represent populations of 2000–8000 and approximate neighborhoods.

### Bike Score data

We obtained Bike Score data shapefiles (2012) for nine Canadian cities and 15 US cities directly from Walk Score (now RedFin Real Estate). We were provided with point files (100 m grid) with attributes for Bike Score and each of its components for each city that was included in the original Bike Score launch. The methodology is here: https://www.walkscore.com/bike-score-methodology.shtml. In brief, Bike Score ranges from 0 to 100, and is comprised of 3 environmental components: a Bike Lane Score, a Hill Score, and a Destinations and Connectivity Score, each ranging from 0 to 100 where 100 describes the most bikeable (more bicycle facilities, flat topography, and more destinations and connectivity, respectively). The Bike Lane Score is derived from cycling infrastructure data provided by municipal governments. It captures painted bicycle lanes, off-street trails, cycle tracks, and residential bikeways; it does not include sharrows (shared-lane markings), or other cycling initiatives such as bicycle parking or bike share programs. The score weights separated facilities twice as much as on-street facilities, and uses a distance decay function to favour proximity. The Hill Score is based on the steepest grade in a 200 m radius area. The Connectivity Score is equivalent to Street Smart Walk Score®. For US cities, the public version of Bike Score also includes a fourth “social” component: the proportion of the population that cycles to work. Given that our aim was to assess how Bike Score predicts cycling behavior, we used the 3 component version of Bike Score for both Canadian and US cities. The summary metric, Bike Score, is a weighted sum of the Bike Lane Score (50 %), Hill Score (25 %), and Destinations and Connectivity Score (25 %). As indicated above, the authors (MW, KT, MB) contributed to the development of Bike Score.

### Spatial summary of Bike Score data

For city level analyses, we used the mean city-wide Bike Score as provided by Walk Score. For within-city analyses, we used ArcGIS 10.2 to calculate a mean Bike Score for each census tract from Bike Score shapefiles. In brief, we imported the Bike Score point data for each city into ArcGIS and merged it into one file per country. We obtained census geography files for Canadian (2011) and US (2010) census tracts, and developed a ModelBuilder toolbox to summarize Bike Score and component score values for each census tract. This process involved (1) attributing each Bike Score point to the appropriate census tract and calculating the average Bike Score for each census tract and (2) calculating the coverage of Bike Score data for each census tract (area with Bike Score data/total area of census tract; using Mollweide projection). We excluded census tracts where Bike Score data coverage was less than 80 % by area.

### Cycling mode share data

We sought to use the most accurate, comparable and up-to-date sources of cycling data. For Canada, we extracted journey to work mode share data from the 2011 National Household Survey, [24] available at both city level and census tract level. For the US, we drew data from American Community Survey, [25] using the 2013 1-year estimates for city level analyses, and 5-year estimates (2012) for census tract analyses. While census journey to work data is the best available, it must be noted that it represents only cycling trips for work purposes, it does not capture multi-modal trips, and the spatial information is linked to the residential location (not route or destination).

### Statistical analysis

All statistical analyses were conducted in R (Additional file 1 includes R code and output) [26]. In the city level analysis we report correlations using both Pearson r and and Spearman ρ, and use linear regression to examine the relationship between Bike Score and cycling mode share. Within each city there was substantial variability in both Bike Score and cycling mode share, and thus we conducted census tract level analyses to understand the relationship at the neighborhood-level. We calculated descriptive statistics across all census tracts and stratified by city.

We used linear regression to analyse the association between Bike Score and cycling mode share, with census tract as the unit of analysis. We calculated unadjusted associations between Bike Score, or its components, and cycling mode share. Given the wide range of Bike Score and its components (0–100), we present coefficients for the effect of a 10-unit change on cycling mode share, and also for Bike Score in five categories (0 to 25, >25 to 50, >50 to 75, >75 to 90, and >90 to 100), as has been used in previous work [11, 13]. We report adjusted associations from fixed effect regression models, with a dummy variable for city. The fixed effect approach accounts for the clustered nature of census tracts within cities, and provides an average estimate of the association across all census tracts in all cities. We also ran city-specific models to provide city-specific estimates of the association, which may be of value to future studies focused on a particular location. We first modeled the outcome of cycling mode share with Bike Score and city as independent variables (model 1). In model 2, the Bike Score components (Bike Lane Score, Hill Score, Destinations and Connectivity Score) and city were independent variables. In model 3, we included the categorized Bike Score variable and city as independent variables. We used New York City as the reference city for all models, given that it has the largest number of census tracts and the lowest mean cycling mode share across census tracts. Finally, we also performed a multilevel model of the association between Bike Score and cycling modes share, using random slope models which allow for different magnitudes in the association across cities. We ran null models, considering city, or city and country, as random effects, and related models with Bike Score as a fixed effect.

## Results

### City level analysis

**Fig. 1:**
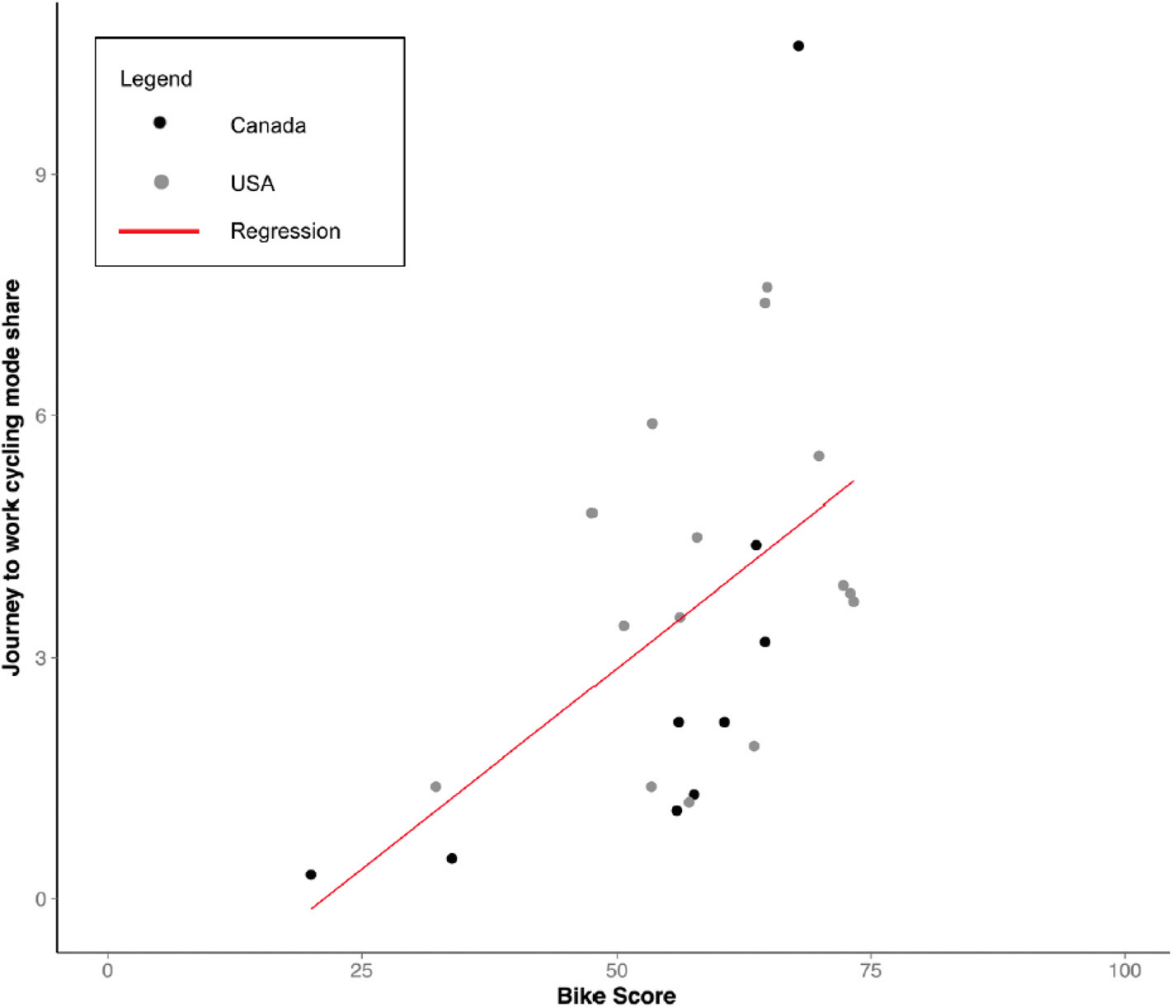
Scatter plot and estimated regression line for City-wide cycling mode sharea and City-wide Average Bike Score®a.^b^ Linear regression estimated association between Bike Score and cycling mode share. We show the full possible range of Bike Score, however, city-wide averages for the study cities do not cover this full range. ^a^City-wide journey to work cycling mode share (% commutes by bike for workers aged 16 years and older) -American Community Survey, 2013 1-year estimates, U.S. Census Bureau; CA data is 2011 National Household Survey, Journey to work Bicycle Mode Share for population aged 15 years and older with a usual place of work. ^b^City-wide Bike Score and components provided directly from the company (now Redfin Real Estate), using 3 components (Bike Lane Score, Hill Score, Destinations and Connectivity Score), May 2012 release

Across the 24 cities, the city-wide mean Bike Scores and mode shares ranged from 20 to 73 and 0.3 to 12.3 %, respectively (Additional file 2: Table S1). In cities with higher mean Bike Score, more people cycled to work (Fig. 1). At the city level, the correlation between mean Bike Score and mean journey to work cycling mode share was moderate (Pearson’s *r* = 0.52 and Spearman’s *ρ* = 0.56). The association between Bike Score and cycling mode share was positive and significant (*β* = 1.5 % for a 10-unit change in Bike Score, 95 % CI 0.4 to 2.6 %) with Bike Score explaining 27 % of the variation in cycling mode share between cities.

### Census tract level analyses

The analysis dataset included 5664 census tracts nested in 24 cities, with a range of 15 (Moncton, New Brunswick) to 2164 (New York City) tracts per city. Across all census tracts, the mean Bike Score was 67.0, with a range from 5.9 to 100. Cycling mode share had a mean of 1.9 % and range of 0.0 to 34.0 %. Table 1 provides the descriptive statistics for cycling mode share and Bike Score (overall and components) stratified by city. Across all census tracts, the correlation between Bike Score and cycling mode share was moderate (Pearson’s *r* = 0.35 and Spearman’s *ρ* = 0.40).

**Table 1:** Descriptive characteristics for 5664 census tracts in 24 study cities

City, State/Province	Number of Census Tracts	Cycling Mode Share^a^ (%) (mean, (SD))	Bike Score®^b^ (mean, (SD))	Bike Lane Score (mean, (SD))	Hill Score (mean, (SD))	Destinations and Connectivity Score (mean, (SD))
Ann Arbor, Michigan	33	3.6 (2.7)	76.4 (13.9)	79.9 (18.2)	91.2 (9.0)	55.6 (26.9)
Austin, Texas	164	1.8 (2.8)	48.3 (17.4)	26.3 (24.5)	85.8 (17.5)	55.3 (27.2)
Boston, Massachusetts	179	1.6 (2.4)	73.4 (19.1)	57.7 (32.0)	88.6 (14.5)	90.4 (17.2)
Calgary, Alberta	221	1.2 (1.8)	74.4 (13.0)	84.0 (19.7)	88.3 (13.3)	42.0 (26.0)
Chicago, Illinois	768	1.2 (2.0)	60.5 (13.6)	25.9 (24.6)	100.0 (0.4)	90.9 (15.0)
Eugene, Oregon	31	10.6 (7.2)	77.9 (18.4)	83.4 (18.4)	82.2 (29.5)	63.6 (24.6)
Fort Collins, Colorado	33	7.8 (6.0)	83.6 (10.7)	93.4 (10.5)	97.8 (5.9)	50.7 (23.8)
Halifax, Nova Scotia	25	3.9 (3.9)	67.4 (14.6)	60.9 (22.2)	71.5 (14.4)	76.9 (22.0)
Madison, Wisconsin	53	5.9 (5.0)	67.4 (19.8)	58.5 (27.5)	91.2 (8.3)	62.0 (26.6)
Minneapolis, Minnesota	115	3.9 (3.3)	77.6 (15.2)	65.8 (27.0)	96.4 (6.3)	82.8 (18.4)
Moncton, New Brunswick	15	0.4 (0.8)	49.3 (15.3)	29.1 (25.6)	94.2 (3.8)	45.5 (30.9)
Montréal, Québec	320	4.8 (4.6)	78.8 (17.7)	64.4 (33.3)	97.8 (9.3)	89.2 (21.9)
New York, New York	2164	0.7 (1.4)	64.8 (18.3)	36.4 (35.7)	95.4 (11.4)	91.6 (19.2)
Portland, Oregon	137	6.3 (5.6)	69.5 (20.3)	58.7 (25.9)	80.5 (26.7)	80.8 (23.1)
San Francisco, California	196	3.1 (3.4)	77.8 (17.3)	84.3 (24.4)	53.8 (32.5)	89.8 (21.1)
Saskatoon, Saskatchewan	45	2.2 (2.4)	78.7 (13.1)	84.5 (20.0)	98.4 (3.3)	48.2 (27.9)
Seattle, Washington	132	3.3 (2.6)	60.9 (19.4)	51.2 (31.8)	65.0 (16.9)	77.1 (25.4)
St. John’s, Newfoundland and Labrador	26	0.0 (0.0)	44.8 (16.7)	30.9 (24.9)	62.2 (23.0)	55.9 (33.3)
Tempe, Arizona	37	4.1 (4.4)	76.2 (12.4)	70.1 (22.8)	99.2 (3.4)	66.2 (15.3)
Toronto, Ontario	544	2.0 (3.8)	66.9 (16.4)	45.7 (30.9)	96.8 (6.4)	80.2 (19.9)
Tucson, Arizona	115	2.6 (3.8)	74.4 (19.2)	72.3 (26.6)	98.8 (5.5)	55.0 (26.7)
Vancouver, British Columbia	115	4.1 (3.7)	78.0 (14.8)	71.2 (27.4)	79.3 (15.2)	91.1 (13.6)
Victoria, British Columbia	17	11.5 (4.3)	74.3 (17.1)	54.2 (32.4)	95.6 (6.2)	93.8 (5.7)
Washington, DC	179	2.5 (3.0)	66.5 (20.9)	52.2 (33.6)	79.5 (19.0)	82.8 (20.0)
Total	5664	1.9 (3.3)	67.0 (18.5)	46.5 (35.7)	91.9 (16.6)	83.6 (24.3)

^a^Census tract level Journey to work Bicycle Mode Share (% commute by bike for workers aged 16 years and older) -American Community Survey, 5-year estimates (2012 5-year estimates), U.S. Census Bureau, 2013 American Community Survey; CA data is 2011 National Household Survey, Census tract level Journey to work Bicycle Mode Share for population aged 15 years and older with a usual place of work

^b^Bike Score spatial data provided from Walk Score (May 2012 release); analysis here includes 3 components (Bike Lane Score, Hill Score, Destinations and Connectivity Score); spatial data aggregated to the census tract in ArcGIS 10.2

In unadjusted analyses, Bike Score and each of the Bike Score components - Bike Lane Score, Hill Score, Destinations and Connectivity Score - were significantly associated with cycling mode share (Table 2). The unadjusted association for Hill Score (higher scores mean flatter topography, which would be hypothesized to promote cycling) was negative - opposite to expectation.

**Table 2:** Results of linear regression models estimating associations between Bike Score® and components^a^, and cycling mode share^b^

		Model 1	Model 2	Model 3
	Unadjusted estimates	Bike score + City Term	Bike Score Components +	Bike Score Categorical
			City Term	
	β (95 % CI)	β (95 % CI)	β (95 % CI)	β (95 % CI)
Intercept		−2.6 (-2.9 to -2.3)	−4.4 (−5.1 to −3.8)	−1.4 (−2.4 to −0.3)
Bike Score (10-unit change)	**0.6 (0.6 to 0.7)**	**0.5 (0.5 to 0.6)**		
Destinations/Connectivity Score (10-unit change)	**0.2 (0.1 to 0.2)**		**0.4 (0.3 to 0.4)**	
Bike Lane Score (10-unit change)	**0.3 (0.3 to 0.3)**		**0.2 (0.2 to 0.2)**	
Hill Score (10-unit change)	**−0.1 (−0.2 to −0.1)**		**0.1 (0.1 to 0.2)**	
Bike Score (categorical)				
0 to 25	0 (Reference)			0 (Reference)
>25 to 50	**−0.2 (−1.3 to 1.0)**			**1.1 (0.1 to 2.2)**
>50 to 75	**0.8 (−0.3 to 2.0)**			**1.8 (0.8 to 2.9)**
>75 to 90	**2.0 (0.8 to 3.1)**			**2.6 (1.5 to 3.6)**
>90 to 100	**3.5 (2.3 to 4.7)**			**4.0 (2.9 to 5.0)**
City				
New York, New York		Reference	Reference	Reference
Ann Arbor, Michigan		**2.3 (1.3 to 3.2)**	**3.4 (2.5 to 4.4)**	**2.5 (1.5 to 3.4)**
Austin, Texas		**2.0 (1.5 to 2.4)**	**2.8 (2.3 to 3.2)**	**1.8 (1.3 to 2.2)**
Boston, Massachusetts		0.5 (0.4 to 0.9)	0.6 (0.2 to 1.0)	0.5 (0.1 to 0.9)
Calgary, Alberta		0.0 (−0.4 to 0.4)	1.5 (1.0 to 1.9)	0.3 (−0.1 to 0.6)
Chicago, Illinois		0.7 (0.5 to 0.9)	0.7 (0.5 to 0.9)	0.7 (0.5 to 1.0)
Eugene, Oregon		**9.3 (8.3 to 10.2)**	**10.3 (9.3 to 11.2)**	**9.4 (8.4 to 10.3)**
Fort Collins, Colorado		**6.2 (5.3 to 7.1)**	**7.5 (6.6 to 8.4)**	**6.3 (5.3 to 7.2)**
Halifax, Nova Scotia		**3.1 (2.0 to 4.2)**	**3.7 (2.6 to 4.8)**	**3.3 (2.2 to 4.4)**
Madison, Wisconsin		**5.0 (4.3 to 5.8)**	**5.9 (5.1 to 6.6)**	**5.1 (4.4 to 5.8)**
Minneapolis, Minnesota		**2.6 (2.0 to 3.1)**	**3.0 (2.5 to 3.5)**	**2.7 (2.2 to 3.2)**
Moncton, New Brunswick		0.5 (−0.9 to 1.8)	**1.5 (0.1 to 2.9)**	0.3 (−1.1 to 1.6)
Montréal, Québec		**3.4 (3.1 to 3.7)**	**3.7 (3.3 to 4.0)**	**3.4 (3.1 to 3.8)**
Portland, Oregon		**5.4 (4.9 to 5.9)**	**5.8 (5.4 to 6.3)**	**5.4 (5.0 to 5.9)**
San Francisco, California		**1.7 (1.3 to 2.1)**	**2.1 (1.6 to 2.5)**	**1.8 (1.4 to 2.2)**
Saskatoon, Saskatchewan		0.8 (0.0 to 1.6)	**2.1 (1.3 to 3.0)**	1.0 (0.2 to 1.8)
Seattle, Washington		**2.8 (2.4 to 3.3)**	**3.3 (2.8 to 3.8)**	**2.8 (2.3 to 3.3)**
St. John’s, Newfoundland and Labrador		0.3 (−0.8 to 1.4)	1.1 (−0.2 to 2.1)	10.6 (−0.2 to 21.4)
Tempe, Arizona		**2.8 (1.9 to 3.7)**	**3.6 (2.8 to 4.5)**	**3.1 (2.2 to 3.9)**
Toronto, Ontario		**1.2 (1.0 to 1.5)**	**1.5 (1.3 to 1.8)**	**1.3 (1.0 to 1.5)**
Tucson, Arizona		**1.4 (0.9 to 1.9)**	**2.4 (1.9 to 3.0)**	**1.4 (0.9 to 1.9)**
Vancouver, British-Columbia		**2.8 (2.3 to 3.3)**	**3.0 (2.5 to 3.6)**	**2.9 (2.4 to 3.4)**
Victoria, British-Columbia		**10.4 (9.1 to 11.6)**	**10.4 (9.2 to 11.7)**	**10.3 (9.0 to 11.6)**
Washington, DC		**1.7 (1.3 to 2.2)**	**2.1 (1.6 to 2.5)**	**1.7 (1.3 to 2.1)**
Adj-R2	0.12 (Bike Score, unadjusted)	**0.35**	**0.36**	**0.34**
AIC	12627	**11035**	**10870**	

Data for 5664 Census Tracts in 24 Cities. Coefficients represent % mode share. Boldface indicates statistical significance (*p* < 0.05)

^a^Bike Score spatial data provided from Walk Score (May 2012 release); analysis here includes 3 components (Bike Lane Score, Hill Score, Destinations and Connectivity Score); spatial data aggregated to the census tract in ArcGIS

^b^Census tract level Journey to work Bicycle Mode Share (% commute by bike for workers aged 16 years and older) -American Community Survey, 5-year estimates (2012 5-year estimates), U.S. Census Bureau, 2013 American Community Survey; CA data is 2011 National Household Survey, Census tract level Journey to work Bicycle Mode Share for population aged 15 years and older with a usual place of work

In multiple linear regression adjusting for city (Table 2, Model 1), a ten-unit increase in the Bike Score of a census tract was associated with a 0.5 % increase in the proportion of population cycling to work (*β* = 0.5, 95 % CI: 0.5 to 0.6). Adjusting for city improved the model fit (Model 1 AIC = 11035, versus unadjusted model AIC = 12627; R^2^ = 0.35 versus R^2^ = 0.12). In the model including all Bike Score components (Table 2, Model 2) each of the components had significant associations and the Hill Score was positively associated with cycling mode share, as expected. The change in the direction between unadjusted and adjusted models suggests confounding of the association by one or more of the other variables in the model (Destinations and Connectivity Score, Bike Lane Score, city). Across the three components, the Destination and Connectivity Score had the largest adjusted estimate (*β* = 0.4, 95 % CI: 0.3 to 0.4), suggesting a slightly stronger relationship with journey to work mode share as compared with the Bike Lane Score (*β* = 0.2, 95 % CI: 0.2 to 0.2) or Hill Score (*β* = 0.1, 95 % CI: 0.1 to 0.2).

**Fig. 2:**
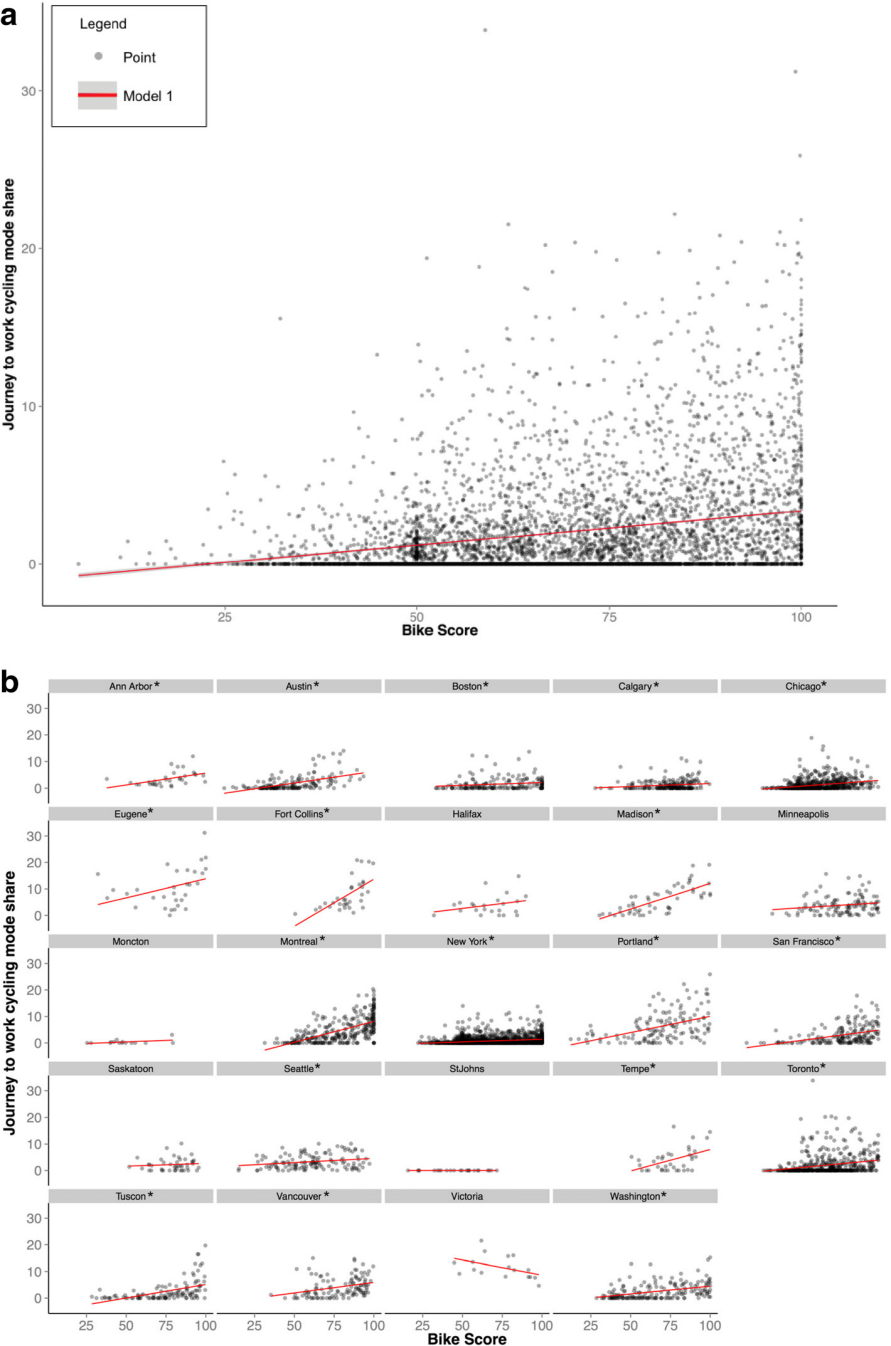
Scatter plot and estimated regression line for cycling mode share^a^ and Bike Score^b^, for 5664 census tracts in 24 study cities (Panel **a**), and stratified by city (Panel **b**) * City-specific regressions with significant slope estimates (see Table 3 for estimate values). **a** Census tract level Journey to work Bicycle Mode Share (% commutes by bike for workers aged 16 years and older) -American Community Survey, 5-year estimates (2012 5-year estimates), U.S. Census Bureau, 2013 American Community Survey; CA data is 2011 National Household Survey, Census tract level Journey to work Bicycle Mode Share for population aged 15 years and older with a usual place of work. **b** Bike Score spatial data provided from Walk Score (May 2012 release); analysis here includes 3 components (Bike Lane Score, Hill Score, Destinations and Connectivity Score); spatial data aggregated to the census tract in ArcGIS 10.2

Across all census tracts there was substantial variability in cycling mode share across Bike Score values (Fig. 2a): there were census tracts with 0 % mode share across all Bike Score values, and at the maximum Bike Score, there were census tracts with mode shares ranging from 0 to above 20 %. Moreover, the distribution of Bike Score was not normal (median = 65, 10^th^ percentile = 46; 90^th^ percentile = 94), and the scatterplot indicated a ceiling effect (140/5664 census tracts have a Bike Score of 100).

Given the potential for non-linear effects we also categorized the Bike Score into five categories, reflecting visual breaks in the data. This model (Table 2, Model 3) showed consistent increases in cycling mode share across the increasing categories of Bike Score. Compared with census tracts with Bike Scores of 0–25, those with Bike Scores of >75 to 90 had mode shares 2.6 % higher (*β* = 2.6, 95 % CI: 1.5 to 3.6), and the highest Bike Score census tracts (>90 to 100) had mode shares 4.0 % higher (*β* = 4.0, 95 % CI: 2.9 to 5.0).

Figure 2b shows city-specific scatterplots and regression lines, highlighting differences in the underlying data and the nature of the association between Bike Score and cycling mode share. Certain cities have no census tracts with low Bike Scores (e.g., Tempe; Saskatoon, Victoria), while others have none with high Bike Scores (e.g., Moncton, St John’s). The strength of the association varies between cities, with cities such as Madison and Fort Collins showing steeper gradients. City-specific regression coefficients (Table 3) were significant for 18 of 23 cities (the model for St John’s had no fit), ranging from a high of a 3.5 % change in mode share for 10 unit change in Bike Score (Fort Collins), to a low of 0.2 % (Boston, Calgary, and New York).

**Table 3:** City-specific linear regression results for cycling mode share and Bike Score (5664 census tracts in 24 study cities)

	Intercept	Bike score coefficient	Adjusted R^2^
		(10-unit change)	
		β (95 % CI)	
Ann Arbor, Michigan	−3.3	**0.9 (0.3–1.5)**	0.19
Austin, Texas	−2.5	**0.9 (0.7–1.1)**	0.30
Boston, Massachusetts	0	**0.2 (0.0–0.4)**	0.02
Calgary, Alberta	−0.5	**0.2 (0.0–0.4)**	0.02
Chicago, Illinois	−1.6	**0.4 (0.3–0.5)**	0.09
Eugene, Oregon	−0.5	**1.4 (0.1–2.7)**	0.10
Fort Collins, Colorado	−21.8	**3.5 (2.0–5.0)**	0.38
Halifax, Nova Scotia	−1	0.7 (−0.4−1.8)	0.04
Madison, Wisconsin	−7.2	**1.9 (1.4–2.4)**	0.55
Minneapolis, Minnesota	0.9	0.4 (0.0–0.8)	0.02
Moncton, New Brunswick	−0.8	0.2 (−0.1−0.5)	0.13
Montréal, Québec	−7.7	**1.6 (1.4–1.8)**	0.36
New York, New York	−0.6	**0.2 (0.2–0.2)**	0.07
Portland, Oregon	−2.3	**1.2 (0.8–1.6)**	0.20
San Francisco, California	−3.2	**0.8 (0.5–1.1)**	0.15
Saskatoon, Saskatchewan	0.5	0.2 (−0.5−0.9)	−0.01
Seattle, Washington	1.4	**0.3 (0.1–0.5)**	0.05
St. John’s, Newfoundland and Labrador	No fit^a^	-	-
Tempe, Arizona	−8.3	**1.6 (0.6–2.6)**	0.19
Toronto, Ontario	−1.8	**0.6 (0.4–0.8)**	0.06
Tucson, Arizona	−5	**1.0 (0.7–1.3)**	0.24
Vancouver, British-Columbia	−2.1	**0.8 (0.4–1.2)**	0.09
Victoria, British-Columbia	19.9	−1.1 (−2.2−0.0)	0.15
Washington, DC	−1.4	**0.6 (0.4–0.8)**	0.16

Bold indicates coefficient is statistically significant at *p* < 0.05

^a^Cycling mode share was 0 % for all census tracts in St. John’s

As we were primarily interested in interpretability and the magnitude of the association (versus its variance) we have focused on the fixed effect models. However, we did also fit multilevel models. These showed similar magnitude in the association between Bike Score and cycling mode share (Additional file 3: Table S2). When we compared the relative fit of multilevel models using the likelihood ratio test between nested models, and found that random slope models fit better than random intercept models.

## Discussion

The development of Bike Score created the first opportunity to conduct between and within city comparisons between cycling mode share and a widely available metric for measuring the cycling environment. Across 24 cities, there was a moderate correlation between Bike Score and journey to work mode share. Prior ecological studies have looked at the environmental, climate, and social influences associated with cycling mode share using cities or health regions as the unit of analysis [27–29], but these may mask important variability in cycling rates and conditions within cities. Given the high resolution Bike Score data we were able to do a census tract level analysis and found that a 10 unit increase in Bike Score was associated with a 0.5 % increase in journey to work mode share, a meaningful difference given the low cycling mode shares across much of North America. This work confirms that Bike Score is associated with cycling mode share, and suggests this metric has utility for research and practice to aid with planning bicycle infrastructure and increasing bicycle mode share.

We found a significant association across all cities, however, our within-city analysis identified important nuances on the association for specific cities. Eighteen cities had significant associations between mode share and Bike Score with estimates varying from modest (0.2 % per 10 unit change in Bike Score) to dramatic (3.5 %) in city-specific models. We conclude that Bike Score shows utility for national or multicity studies, but closer inspection may be needed prior to its application for city-specific analysis and planning in certain locations.

The development of Bike Score was based on environmental factors consistently related to cycling in the literature [19, 30]. We found that Bike Lane Score, Hill Score, and the Destinations and Connectivity Score were all independently associated with cycling mode share. The Destination and Connectivity Score had a marginally stronger association than the other components in the adjusted model (Model 2). This score is equivalent to the Walk Score’s ‘Street Smart Walk Score’, and thus this finding highlights synergies between promoting walking and cycling. Topography is arguably more of a barrier for cycling than for walking. The study cities included very hilly areas (San Francisco, Seattle) and also very flat areas (Saskatoon), and the Hill Score maintained an independent effect: areas with fewer hills had higher cycling mode shares in the adjusted model.

Of the three components, the Bike Lane Score may be the most actionable component for local and regional governments in the short term. The importance of cycling-specific facilities has been emphasized for promoting safe and comfortable cycling [21] as well as attracting new cyclists [31]. The data for the Bike Lane Score was provided directly from city governments, with only the following cycling infrastructure included: bike lanes, residential street bikeways (combined as on-street), and cycle tracks and off-street paths (combined as off-street). The Bike Lane Score could be considered an indicator of safety, given that these infrastructure types are safer than major streets [32, 33] and are preferred by cyclists, [34] especially women and those new to bicycling [31, 35]. Subsequent work may evaluate correlations between Bike Score and cycling safety, although obtaining consistent and comparable safety data across countries, cities and census tracts will be a challenge.

The metrics developed by Walk Score (Walk Score, Bike Score, and Transit Score) are intuitive, easy to use, and available online, fulfilling many of the recommendations for making built environment measures relevant to practice [36]. The Bike Score methodology was informed by empirical research, however, the specific algorithms and decay functions are proprietary. We recommended that users think critically about the quality of the underlying data sources. The Hill Score is based on the widely-used National Elevation Data set from the US Geological Survey [37]. The Bike Lane Score is based on data provided by local governments in 2012 and again in 2015 according to standardized criteria. In the future bicycle facility data may be derived from open sources (e.g., Open Street Maps) although this brings concern around consistency across cities. The Destination and Connectivity Score is the Street Smart Walk Score, for which destinations are identified using a proprietary search strategy across diverse databases. We cannot know if there is spatial bias in the completeness of the amenity data. The metrics are constantly updated, a strength for current research but a challenge for longitudinal studies. Researchers should ensure they report the calculation date for the data used, and in the long term, data archiving may be needed. We observed that Bike Score also has a ceiling effect (many census tracts scoring high), so that Bike Score may have limited sensitivity for tracking change in already bikeable areas. Similarly, as Bike Score was created based on North American cities. Given the relatively low prevalence of cycling infrastructure in many North American cities, the score may not be calibrated for other locations, especially those with extensive infrastructure. Finally to note that we used a Bike Score including the three environmental components, to analyse how this predicts mode share; data for all components are available from the company, but the version of Bike Score visible on the website for US cities includes the mode share component.

This is the first study to use Bike Score, and covers 24 cities across two countries. Several limitations of the current work should be acknowledged. We used journey to work mode share data from national surveys, the only comparable data across study cities. For the American Community Survey, we used 2012 5-year averages to increase the stability of the estimates for census tracts. We used the 2011 Canadian National Household Survey for temporal alignment, but 5-year means do not exist. In addition, this is a voluntary survey and may carry higher non-response error than the Census data that was formerly available. Journey to work mode share data is spatially located to work trip origins (home locations), and is not necessarily an indicator of the areas with the highest cycling volumes or destinations. Of note, the 24 study cities included here are those in the initial Bike Score launch; while the Canadian cities are diverse, many of the US cities were selected because they had high cycling rates. The 9 Canadian cities included comprise 6.7 million people, or 20.1 % of the Canadian population, whilst the 15 US cities comprise 16.6 million people, or 2.1 % of the US population. The included cities have great variety in terms of size and climate (Additional file 2: Table S1). Subsequent research may investigate how neighbourhood composition impacts associations between Bike Score and cycling mode share. The city-wide Bike Score used in this analysis also includes some proprietary population weighting, which explains why the city-wide mean Bike Scores do not match with the mean of the census tracts for that city. City-wide averages are also sensitive to administrative city boundaries, which include surrounding suburbs in some cases but not others, and it is possible that these may differ between Canadian and US cities. Finally, this is a cross-sectional analysis of a new metric. Planners can use Bike Score to prioritize where to locate new infrastructure, and subsequent research may assess if changes in Bike Score are associated with changes in mode share.

## Conclusions

The new Bike Score index predicts some of the variability in cycling to work mode share, and can be used for research with similar utility to the popular Walk Score metric [15, 16, 11]. Given the demonstrated significant and meaningful association across neighborhoods in diverse US and Canadian cities, Bike Score may be a valuable tool to aid with research and with planning for bicycle infrastructure and increasing bicycle mode in large studies. Further, our city-specific analyses showed some city level variation, suggesting that studies within a city should further investigate the suitability of this score and its component scores for their setting.

## Additional files

Additional file 1:
**R code and output for all analyses.** (PDF 1350 kb) Additional file 2: Table S1. Characteristics of 24 study cities (city-level variables). (DOCX 15 kb) Additional file 3: Table S2. Results of linear multilevel models. (DOCX 13 kb)
