# Supplementary material for: Bike Score®: Associations between urban bikeability and cycling behavior in 24 cities
Source: Int J Behav Nutr Phys Act. 2016 Feb 11;13:18. doi: 10.1186/s12966-016-0339-0 (PMC4751700; doi:10.1186/s12966-016-0339-0)
Supplement: Additional file 1: — R code and output for all analyses. (PDF 1350 kb) [file 12966_2016_339_MOESM1_ESM.pdf]

Bike Score Paper Analysis

Meghan Winters - Simon Fraser University Kay Teschke - University of British Columbia Michael Brauer - University of British Columbia Daniel Fuller - University of Saskatchewan ————————-

Importing Bike Score Data

Pre importing steps

1. Convert the EXCEL file to CSV

Importing the data.

```
bikeScore1<-read.csv("/Users/DogLeg/Dropbox/Data/BikeScore/BikeScore_CensusUnits_2012UPDATED.csv")
```

Generating some descriptive statistics for each variable

Variables labels are as follows:

1. Outcome: *pc_bike_2012_2012 (journey-to-work mode share)
2. Average Bike Score:

- avg_bikesc (range 0-100, average bike score)

3. Components of Bike Score:

- avg_bikela (bike lanes)
- avg_roughness (topography)
- avg_stret (streetsmart walkscore, i.e., destinations)

4. Cluster:

- city

5. Ignore

- avg_degree

Recoding long string variables to something manageable in R

1. Replaced the cities names with sorting strings with no special characters

- Montréal to Montreal
- Minneapolis_Hennapin_county_MN to Minneapolis_MN
- Fort_Collins

```
bikeScore1 <- as.data.frame(sapply(bikeScore1,gsub,pattern="Montr\x8eal",replacement="Montreal"))
bikeScore1 <- as.data.frame(sapply(bikeScore1,gsub,pattern="Fort_Collins_Larimer_County",replacement="Fort Collins"))
bikeScore1 <- as.data.frame(sapply(bikeScore1,gsub,pattern="Minneapolis_Hennapin_county_MN",replacement="Minneapolis"))
bikeScore1 <- as.data.frame(sapply(bikeScore1,gsub,pattern="Ann_Arbor",replacement="Ann Arbor"))
bikeScore1 <- as.data.frame(sapply(bikeScore1,gsub,pattern="Austin_TX",replacement="Austin"))
bikeScore1 <- as.data.frame(sapply(bikeScore1,gsub,pattern="Boston_MA",replacement="Boston"))
bikeScore1 <- as.data.frame(sapply(bikeScore1,gsub,pattern="Chicago_IL",replacement="Chicago"))
bikeScore1 <- as.data.frame(sapply(bikeScore1,gsub,pattern="Eugene_OR",replacement="Eugene"))
bikeScore1 <- as.data.frame(sapply(bikeScore1,gsub,pattern="Madison_WI",replacement="Madison"))
bikeScore1 <- as.data.frame(sapply(bikeScore1,gsub,pattern="Minneapolis_MN",replacement="Minneapolis"))
bikeScore1 <- as.data.frame(sapply(bikeScore1,gsub,pattern="NY_NY",replacement="New York"))
bikeScore1 <- as.data.frame(sapply(bikeScore1,gsub,pattern="Portland_OR",replacement="Portland"))
bikeScore1 <- as.data.frame(sapply(bikeScore1,gsub,pattern="SanFran",replacement="San Francisco"))
bikeScore1 <- as.data.frame(sapply(bikeScore1,gsub,pattern="Seattle_WA",replacement="Seattle"))
bikeScore1 <- as.data.frame(sapply(bikeScore1,gsub,pattern="Tempe_AZ",replacement="Tempe"))
bikeScore1 <- as.data.frame(sapply(bikeScore1,gsub,pattern="Tucson_AZ",replacement="Tuscon"))
bikeScore1 <- as.data.frame(sapply(bikeScore1,gsub,pattern="Washington_DC",replacement="Washington"))
bikeScore1 <- as.data.frame(sapply(bikeScore1,gsub,pattern="St. John's",replacement="StJohns"))
```

Recoding variables as numeric instead of factor (i.e., categorical)

```
bikeScore1$pc_bike_2012 <- as.numeric(as.character(bikeScore1$pc_bike_2012))
bikeScore1$avg_bikesc <- as.numeric(as.character(bikeScore1$avg_bikesc))
bikeScore1$avg_bikela <- as.numeric(as.character(bikeScore1$avg_bikela))
bikeScore1$avg_roughn <- as.numeric(as.character(bikeScore1$avg_roughn))
bikeScore1$avg_stret <- as.numeric(as.character(bikeScore1$avg_stret))
bikeScore1$avg_degree <- as.numeric(as.character(bikeScore1$avg_degree))
```

Importing City Level Data

Pre importing steps

1. Convert the EXCEL file to CSV

Importing the data.

```
cityData<-read.csv("/Users/DogLeg/Dropbox/Data/BikeScore/BikeScoreCityLevelVariablesFinal.csv")
```

Generating some descriptive statistics for each variable

Variables labels are as follows:

1. citypop:

- Population of the City

2. citypopdenssqkm:

- City Population Density by Square Kilometer

3. AverageTempJan:

- Average January Temperature in Celcuis

4. AverageTempJul:

- Average July Temperature in Celcuis

5. TotalWetDays

- Total number of days with rain in a year

Recoding long string variables to something manageable in R

1. Replaced the cities names with sorting strings with no special characters

- Montréal to Montreal
- Minneapolis_Hennapin_county_MN to Minneapolis_MN
- Fort_Collins

```
cityData <- as.data.frame(sapply(cityData,gsub,pattern="Montr_al",replacement="Montreal"))
cityData <- as.data.frame(sapply(cityData,gsub,pattern="Fort_Collins_Larimer_County",replacement="Fort Collins"))
cityData <- as.data.frame(sapply(cityData,gsub,pattern="Minneapolis_Hennapin_county_MN",replacement="Minneapolis"))
cityData <- as.data.frame(sapply(cityData,gsub,pattern="Ann_Arbor",replacement="Ann Arbor"))
cityData <- as.data.frame(sapply(cityData,gsub,pattern="Austin_TX",replacement="Austin"))
cityData <- as.data.frame(sapply(cityData,gsub,pattern="Boston_MA",replacement="Boston"))
cityData <- as.data.frame(sapply(cityData,gsub,pattern="Chicago_IL",replacement="Chicago"))
cityData <- as.data.frame(sapply(cityData,gsub,pattern="Eugene_OR",replacement="Eugene"))
cityData <- as.data.frame(sapply(cityData,gsub,pattern="Madison_WI",replacement="Madison"))
cityData <- as.data.frame(sapply(cityData,gsub,pattern="Minneapolis_MN",replacement="Minneapolis"))
cityData <- as.data.frame(sapply(cityData,gsub,pattern="NY_NY",replacement="New York"))
cityData <- as.data.frame(sapply(cityData,gsub,pattern="Portland_OR",replacement="Portland"))
cityData <- as.data.frame(sapply(cityData,gsub,pattern="SanFran",replacement="San Francisco"))
cityData <- as.data.frame(sapply(cityData,gsub,pattern="Seattle_WA",replacement="Seattle"))
cityData <- as.data.frame(sapply(cityData,gsub,pattern="Tempe_AZ",replacement="Tempe"))
cityData <- as.data.frame(sapply(cityData,gsub,pattern="Tucson_AZ",replacement="Tuscon"))
cityData <- as.data.frame(sapply(cityData,gsub,pattern="Washington_DC",replacement="Washington"))
cityData <- as.data.frame(sapply(cityData,gsub,pattern="St. John's",replacement="StJohns"))
```

Recoding variables as numeric instead of factor (i.e., categorical)

```
cityData$citypopdenssqkm <- as.numeric(as.character(cityData$citypopdenssqkm))
cityData$citypop <- as.numeric(as.character(cityData$citypop))
cityData$AverageTempJan <- as.numeric(as.character(cityData$AverageTempJan))
cityData$AverageTempJul <- as.numeric(as.character(cityData$AverageTempJul))
cityData$TotalWetDays <- as.numeric(as.character(cityData$TotalWetDays))
```

Merging City Level Data

```
bikeScore <- merge(bikeScore1, cityData, by.x=c("city"), by.y=c("City"))
write.csv(bikeScore, "/Users/DogLeg/Dropbox/Data/BikeScore/BikeScore_Clean.csv")
```

Table 1. Descriptives

```
sumfun <- function(x, ...){
  c(m=mean(x, ...), Stand_Dev=sd(x, ...), l=length(x))}
library(doBy)
```

```
## Loading required package: survival
```

```
library(xtable)
popcity <- summaryBy(pc_bike_2012 + avg_bikesc + avg_bikela + avg_roughn + avg_stret + Country ~ city, data = bikeScore, FUN =
sumfun, na.rm=TRUE)
print(xtable(popcity), type = "html", )
```

	city	pc_bike_2012.m	pc_bike_2012.Stand_Dev	pc_bike_2012.l	avg_bikesc.m	avg_bikesc.Stand_Dev	avg_bikesc.l	avg_bikela.m	avg_bikela.Stand_Dev
1	Ann Arbor	3.59	2.68	33.00	76.43	13.87	33.00	79.85	18.15
2	Austin	1.82	2.80	164.00	48.28	17.40	164.00	26.30	24.46
3	Boston	1.58	2.38	179.00	73.44	19.07	179.00	57.66	31.97
4	Calgary	1.18	1.83	221.00	74.36	12.99	221.00	84.04	19.72
5	Chicago	1.17	2.01	768.00	60.46	13.59	768.00	25.90	24.55
6	Eugene	10.64	7.21	31.00	77.94	18.43	31.00	83.38	18.35
7	Fort Collins	7.82	5.98	33.00	83.58	10.65	33.00	93.43	10.50
8	Halifax	3.91	3.86	25.00	67.42	14.57	25.00	60.85	22.18
9	Madison	5.87	5.04	53.00	67.39	19.79	53.00	58.45	27.53
10	Minneapolis	3.88	3.32	115.00	77.57	15.16	115.00	65.84	26.99
11	Moncton	0.36	0.82	15.00	49.30	15.32	15.00	29.05	25.62
12	Montreal	4.81	4.63	320.00	78.75	17.70	320.00	64.35	33.32
13	New York	0.69	1.42	2164.00	64.76	18.32	2164.00	36.36	35.67
14	Portland	6.33	5.60	137.00	69.52	20.34	137.00	58.72	25.85
15	San Francisco	3.07	3.43	196.00	77.82	17.28	196.00	84.33	24.41
16	Saskatoon	2.23	2.37	45.00	78.65	13.09	45.00	84.54	19.99
17	Seattle	3.31	2.61	132.00	60.93	19.35	132.00	51.18	31.83
18	StJohns	0.00	0.00	26.00	44.84	16.67	26.00	30.92	24.92
19	Tempe	4.07	4.41	37.00	76.20	12.39	37.00	70.11	22.79
20	Toronto	1.99	3.84	544.00	66.89	16.43	544.00	45.69	30.92
21	Tuscon	2.55	3.81	115.00	74.43	19.15	115.00	72.34	26.62
22	Vancouver	4.14	3.73	115.00	77.96	14.80	115.00	71.15	27.43
23	Victoria	11.52	4.28	17.00	74.27	17.14	17.00	54.24	32.43
24	Washington	2.51	2.98	179.00	66.53	20.92	179.00	52.21	33.64

```
library(psych)
library(Hmisc)
```

```
## Loading required package: grid
## Loading required package: lattice
## Loading required package: Formula
## Loading required package: ggplot2
##
## Attaching package: 'ggplot2'
##
## The following object is masked from 'package:psych':
##
##     %+%
##
##
## Attaching package: 'Hmisc'
##
## The following object is masked from 'package:psych':
##
##     describe
##
## The following objects are masked from 'package:xtable':
##
##     label, label<-
##
## The following objects are masked from 'package:base':
##
##     format.pval, round.POSIXt, trunc.POSIXt, units
```

```
psych::describe(bikeScore$pc_bike_2012)
```

```
##   vars    n mean   sd median trimmed  mad min   max range skew kurtosis
## 1    1 5580 1.94 3.31   0.45    1.16 0.67   0 33.85 33.85 2.77    10.02
##     se
## 1 0.04
```

```
psych::describe(bikeScore$avg_bikesc)
```

```
##   vars    n  mean    sd median trimmed   mad min max range skew kurtosis
## 1    1 5662 66.97 18.52  65.03   66.77 22.28 5.9 100  94.1 0.07    -0.86
##     se
## 1 0.25
```

```
psych::describe(bikeScore$avg_roughn)
```

```
##   vars    n  mean    sd median trimmed  mad min max range  skew kurtosis
## 1    1 5662 91.94 16.56  99.98   96.24 0.03   0 100   100 -2.92     9.32
##     se
## 1 0.22
```

```
psych::describe(bikeScore$avg_stret)
```

```
##   vars    n  mean    sd median trimmed  mad min max range  skew kurtosis
## 1    1 5662 83.64 24.32   98.1   88.82 2.82   0 100   100 -1.56     1.45
##     se
## 1 0.32
```

```
psych::describe(bikeScore$avg_bikela)
```

```
##   vars    n  mean    sd median trimmed   mad min max range skew kurtosis
## 1    1 5662 46.52 35.68  43.56   45.65 50.22   0 100   100 0.16    -1.41
##     se
## 1 0.47
```

```
### Pearson Correlation

cor(bikeScore$avg_bikesc, bikeScore$pc_bike_2012, use="complete.obs", method="pearson")
```

```
## [1] 0.3529364
```

```
### Spearman Correlation

cor(bikeScore$avg_bikesc, bikeScore$pc_bike_2012, use="complete.obs", method="spearman")
```

```
## [1] 0.4034629
```

```
bikescore_100 <- subset(bikeScore, avg_bikesc > 89)
bikescore_10 <- subset(bikeScore, avg_bikesc < 11)
```

City level correlation between BikeScore and Cycling to work

```
rcorr(popcity$avg_bikesc.m, popcity$pc_bike_2012.m)
```

```
##      x    y
## x 1.00 0.52
## y 0.52 1.00
##
## n= 24
##
##
## P
##   x      y
## x        0.0095
## y 0.0095
```

```
cycle_bikescore_city <- lm(pc_bike_2012.m ~ avg_bikesc.m, data=popcity)
summary(cycle_bikescore_city)
```

```
##
## Call:
## lm(formula = pc_bike_2012.m ~ avg_bikesc.m, data = popcity)
##
## Residuals:
##     Min      1Q  Median      3Q     Max
## -3.2464 -1.4727 -0.6979  0.9606  7.1074
##
## Coefficients:
##              Estimate Std. Error t value Pr(>|t|)
## (Intercept)  -6.55174    3.65141  -1.794  0.08652 .
## avg_bikesc.m  0.14767    0.05199   2.840  0.00952 **
## ---
## Signif. codes:  0 '***' 0.001 '**' 0.01 '*' 0.05 '.' 0.1 ' ' 1
##
## Residual standard error: 2.596 on 22 degrees of freedom
## Multiple R-squared:  0.2683, Adjusted R-squared:  0.2351
## F-statistic: 8.067 on 1 and 22 DF,  p-value: 0.009521
```

```
confint(cycle_bikescore_city)
```

```
##                     2.5 %    97.5 %
## (Intercept)  -14.12430719 1.0208241
## avg_bikesc.m   0.03984689 0.2554883
```

```
### Canada

city_canada <- subset(bikeScore, Country=="Canada")
popcity_canada <- summaryBy(pc_bike_2012 + avg_bikesc + avg_bikela + avg_roughn + avg_stret ~ city, data = city_canada, FUN = s
umfun, na.rm=TRUE)

### Canada Correlation

rcorr(popcity_canada$avg_bikesc.m, popcity_canada$pc_bike_2012.m)
```

```
##      x    y
## x 1.00 0.52
## y 0.52 1.00
##
## n= 8
##
##
## P
##   x      y
## x        0.1881
## y 0.1881
```

```
### Canada Regression

cycle_bikescore_canada <- lm(pc_bike_2012.m ~ avg_bikesc.m, data=popcity_canada)
summary(cycle_bikescore_canada)
```

```
##
## Call:
## lm(formula = pc_bike_2012.m ~ avg_bikesc.m, data = popcity_canada)
##
## Residuals:
##     Min      1Q  Median      3Q     Max
## -2.9833 -1.4740 -0.3876  0.0204  7.3700
##
## Coefficients:
##              Estimate Std. Error t value Pr(>|t|)
## (Intercept)  -6.40623    6.63629  -0.965    0.372
## avg_bikesc.m  0.14217    0.09575   1.485    0.188
##
## Residual standard error: 3.447 on 6 degrees of freedom
## Multiple R-squared:  0.2687, Adjusted R-squared:  0.1468
## F-statistic: 2.205 on 1 and 6 DF,  p-value: 0.1881
```

```
### USA

city_usa <- subset(bikeScore, Country=="USA")
popcity_usa <- summaryBy(pc_bike_2012 + avg_bikesc + avg_bikela + avg_roughn + avg_stret ~ city, data = city_usa, FUN = sumfun,
na.rm=TRUE)

### USA Correlation

rcorr(popcity_usa$avg_bikesc.m, popcity_usa$pc_bike_2012.m)
```

```
##      x    y
## x 1.00 0.51
## y 0.51 1.00
##
## n= 16
##
##
## P
##   x      y
## x        0.0442
## y 0.0442
```

```
### USA Regression

cycle_bikescore_usa <- lm(pc_bike_2012.m ~ avg_bikesc.m, data=popcity_usa)
summary(cycle_bikescore_usa)
```

```
##
## Call:
## lm(formula = pc_bike_2012.m ~ avg_bikesc.m, data = popcity_usa)
##
## Residuals:
##     Min      1Q  Median      3Q     Max
## -2.8416 -1.4722 -0.8157  1.3611  5.5424
##
## Coefficients:
##              Estimate Std. Error t value Pr(>|t|)
## (Intercept)   -6.6393     4.8139  -1.379   0.1895
## avg_bikesc.m   0.1505     0.0681   2.211   0.0442 *
## ---
## Signif. codes:  0 '***' 0.001 '**' 0.01 '*' 0.05 '.' 0.1 ' ' 1
##
## Residual standard error: 2.335 on 14 degrees of freedom
## Multiple R-squared:  0.2588, Adjusted R-squared:  0.2058
## F-statistic: 4.887 on 1 and 14 DF,  p-value: 0.0442
```

Bivariate linear regression analysis - Census Tract Level

```
biv_avg_bikesc <- lm(pc_bike_2012 ~ avg_bikesc, data=bikeScore)
summary(biv_avg_bikesc)
```

```
##
## Call:
## lm(formula = pc_bike_2012 ~ avg_bikesc, data = bikeScore)
##
## Residuals:
##    Min     1Q Median     3Q    Max
## -4.031 -1.668 -0.816  0.547 32.434
##
## Coefficients:
##              Estimate Std. Error t value Pr(>|t|)
## (Intercept) -2.317919   0.156886  -14.78   <2e-16 ***
## avg_bikesc   0.063492   0.002254   28.17   <2e-16 ***
## ---
## Signif. codes:  0 '***' 0.001 '**' 0.01 '*' 0.05 '.' 0.1 ' ' 1
##
## Residual standard error: 3.1 on 5577 degrees of freedom
##   (85 observations deleted due to missingness)
## Multiple R-squared:  0.1246, Adjusted R-squared:  0.1244
## F-statistic: 793.5 on 1 and 5577 DF,  p-value: < 2.2e-16
```

```r
confint(biv_avg_bikesc)
```

```
##                   2.5 %      97.5 %
## (Intercept) -2.62547701 -2.01036033
## avg_bikesc   0.05907361  0.06791067
```

```r
extractAIC(biv_avg_bikesc)
```

```
## [1]     2.00 12627.03
```

```r
biv_citypop <- lm(pc_bike_2012 ~ citypop, data=bikeScore)
summary(biv_citypop)
```

```
## 
## Call:
## lm(formula = pc_bike_2012 ~ citypop, data = bikeScore)
## 
## Residuals:
##     Min      1Q  Median      3Q     Max 
## -3.2705 -2.3520 -0.5470  0.5596 31.4349 
## 
## Coefficients:
##               Estimate Std. Error t value Pr(>|t|)    
## (Intercept)  3.294e+00  6.524e-02   50.48   <2e-16 ***
## citypop     -3.360e-07  1.248e-08  -26.92   <2e-16 ***
## ---
## Signif. codes:  0 '***' 0.001 '**' 0.01 '*' 0.05 '.' 0.1 ' ' 1
## 
## Residual standard error: 3.117 on 5578 degrees of freedom
##   (84 observations deleted due to missingness)
## Multiple R-squared:  0.115,  Adjusted R-squared:  0.1148 
## F-statistic: 724.6 on 1 and 5578 DF,  p-value: < 2.2e-16
```

```r
confint(biv_citypop)
```

```
##                     2.5 %        97.5 %
## (Intercept)  3.165764e+00  3.421556e+00
## citypop     -3.604463e-07 -3.115083e-07
```

```r
biv_citypopdenssqkm <- lm(pc_bike_2012 ~ citypopdenssqkm, data=bikeScore)
summary(biv_citypopdenssqkm)
```

```
## 
## Call:
## lm(formula = pc_bike_2012 ~ citypopdenssqkm, data = bikeScore)
## 
## Residuals:
##     Min      1Q  Median      3Q     Max 
## -3.6166 -2.1582 -0.7503  0.5275 31.3335 
## 
## Coefficients:
##                   Estimate Std. Error t value Pr(>|t|)    
## (Intercept)      3.684e+00  8.484e-02   43.42   <2e-16 ***
## citypopdenssqkm -2.812e-04  1.189e-05  -23.66   <2e-16 ***
## ---
## Signif. codes:  0 '***' 0.001 '**' 0.01 '*' 0.05 '.' 0.1 ' ' 1
## 
## Residual standard error: 3.159 on 5578 degrees of freedom
##   (84 observations deleted due to missingness)
## Multiple R-squared:  0.09117,    Adjusted R-squared:  0.09101 
## F-statistic: 559.5 on 1 and 5578 DF,  p-value: < 2.2e-16
```

```r
confint(biv_citypopdenssqkm)
```

```
##                         2.5 %        97.5 %
## (Intercept)      3.5172407351  3.8498780797
## citypopdenssqkm -0.0003045562 -0.0002579393
```

```r
biv_AverageTempJan <- lm(pc_bike_2012 ~ AverageTempJan, data=bikeScore)
summary(biv_AverageTempJan)
```

```
##
## Call:
## lm(formula = pc_bike_2012 ~ AverageTempJan, data = bikeScore)
##
## Residuals:
##    Min     1Q Median     3Q    Max
## -2.042 -1.936 -1.492  0.564 31.892
##
## Coefficients:
##                 Estimate Std. Error t value Pr(>|t|)
## (Intercept)     1.937314   0.045231  42.831   <2e-16 ***
## AverageTempJan -0.005961   0.008251  -0.723     0.47
## ---
## Signif. codes:  0 '***' 0.001 '**' 0.01 '*' 0.05 '.' 0.1 ' ' 1
##
## Residual standard error: 3.313 on 5578 degrees of freedom
##   (84 observations deleted due to missingness)
## Multiple R-squared:  9.358e-05,  Adjusted R-squared:  -8.568e-05
## F-statistic: 0.5221 on 1 and 5578 DF,  p-value: 0.47
```

```r
confint(biv_AverageTempJan)
```

```
##                      2.5 %     97.5 %
## (Intercept)     1.84864310 2.02598477
## AverageTempJan -0.02213551 0.01021296
```

```r
biv_AverageTempJul <- lm(pc_bike_2012 ~ AverageTempJul, data=bikeScore)
summary(biv_AverageTempJul)
```

```
##
## Call:
## lm(formula = pc_bike_2012 ~ AverageTempJul, data = bikeScore)
##
## Residuals:
##    Min     1Q Median     3Q    Max
## -3.362 -1.716 -1.306  0.553 31.783
##
## Coefficients:
##                Estimate Std. Error t value Pr(>|t|)
## (Intercept)     6.87301    0.33613   20.45   <2e-16 ***
## AverageTempJul -0.21945    0.01484  -14.79   <2e-16 ***
## ---
## Signif. codes:  0 '***' 0.001 '**' 0.01 '*' 0.05 '.' 0.1 ' ' 1
##
## Residual standard error: 3.25 on 5578 degrees of freedom
##   (84 observations deleted due to missingness)
## Multiple R-squared:  0.03773,    Adjusted R-squared:  0.03756
## F-statistic: 218.7 on 1 and 5578 DF,  p-value: < 2.2e-16
```

```r
confint(biv_AverageTempJul)
```

```
##                     2.5 %     97.5 %
## (Intercept)     6.2140743  7.5319469
## AverageTempJul -0.2485366 -0.1903595
```

```r
biv_TotalWetDays <- lm(pc_bike_2012 ~ TotalWetDays, data=bikeScore)
summary(biv_TotalWetDays)
```

```
##
## Call:
## lm(formula = pc_bike_2012 ~ TotalWetDays, data = bikeScore)
##
## Residuals:
##    Min     1Q Median     3Q    Max
## -1.981 -1.947 -1.497  0.566 31.897
##
## Coefficients:
##              Estimate Std. Error t value Pr(>|t|)
## (Intercept)  1.844587   0.250796   7.355 2.19e-13 ***
## TotalWetDays 0.000847   0.002109   0.402    0.688
## ---
## Signif. codes:  0 '***' 0.001 '**' 0.01 '*' 0.05 '.' 0.1 ' ' 1
##
## Residual standard error: 3.313 on 5578 degrees of freedom
##   (84 observations deleted due to missingness)
## Multiple R-squared:  2.892e-05,  Adjusted R-squared:  -0.0001504
## F-statistic: 0.1613 on 1 and 5578 DF,  p-value: 0.688
```

```
confint(biv_TotalWetDays)
```

```
##                     2.5 %      97.5 %
## (Intercept)   1.352929997 2.336243931
## TotalWetDays -0.003287288 0.004981246
```

Fixed Effects Model

```
fe1<-lm(pc_bike_2012 ~ avg_bikesc + relevel(city, "New York"), data=bikeScore)
summary(fe1)
```

```
##
## Call:
## lm(formula = pc_bike_2012 ~ avg_bikesc + relevel(city, "New York"),
##     data = bikeScore)
##
## Residuals:
##     Min      1Q  Median      3Q     Max
## -10.609  -1.396  -0.368   0.601  32.273
##
## Coefficients:
##                                        Estimate Std. Error t value
## (Intercept)                           -2.628669   0.148610 -17.688
## avg_bikesc                             0.050975   0.002102  24.250
## relevel(city, "New York")Ann Arbor     2.317793   0.471175   4.919
## relevel(city, "New York")Austin        1.980069   0.220815   8.967
## relevel(city, "New York")Boston        0.463330   0.213395   2.171
## relevel(city, "New York")Calgary       0.020722   0.190628   0.109
## relevel(city, "New York")Chicago       0.710380   0.113644   6.251
## relevel(city, "New York")Eugene        9.292826   0.486040  19.119
## relevel(city, "New York")Fort Collins  6.183678   0.472178  13.096
## relevel(city, "New York")Halifax       3.121068   0.562359   5.550
## relevel(city, "New York")Madison       5.037532   0.376569  13.377
## relevel(city, "New York")Minneapolis   2.555907   0.258183   9.900
## relevel(city, "New York")Moncton       0.479055   0.695821   0.688
## relevel(city, "New York")Montreal      3.414131   0.165699  20.604
## relevel(city, "New York")Portland      5.412641   0.236655  22.871
## relevel(city, "New York")San Francisco 1.717703   0.202605   8.478
## relevel(city, "New York")Saskatoon     0.814372   0.414295   1.966
## relevel(city, "New York")Seattle       2.835051   0.240791  11.774
## relevel(city, "New York")StJohns       0.269064   0.552039   0.487
## relevel(city, "New York")Tempe         2.812674   0.445434   6.314
## relevel(city, "New York")Toronto       1.207942   0.129558   9.324
## relevel(city, "New York")Tuscon        1.361434   0.258725   5.262
## relevel(city, "New York")Vancouver     2.796474   0.258268  10.828
## relevel(city, "New York")Victoria     10.366052   0.653466  15.863
## relevel(city, "New York")Washington    1.748020   0.209354   8.350
##                                       Pr(>|t|)
## (Intercept)                            < 2e-16 ***
## avg_bikesc                             < 2e-16 ***
## relevel(city, "New York")Ann Arbor    8.94e-07 ***
## relevel(city, "New York")Austin        < 2e-16 ***
## relevel(city, "New York")Boston         0.0300 *
## relevel(city, "New York")Calgary        0.9134
## relevel(city, "New York")Chicago      4.38e-10 ***
## relevel(city, "New York")Eugene        < 2e-16 ***
## relevel(city, "New York")Fort Collins  < 2e-16 ***
## relevel(city, "New York")Halifax      2.99e-08 ***
## relevel(city, "New York")Madison       < 2e-16 ***
## relevel(city, "New York")Minneapolis   < 2e-16 ***
## relevel(city, "New York")Moncton        0.4912
## relevel(city, "New York")Montreal      < 2e-16 ***
## relevel(city, "New York")Portland      < 2e-16 ***
## relevel(city, "New York")San Francisco < 2e-16 ***
## relevel(city, "New York")Saskatoon      0.0494 *
## relevel(city, "New York")Seattle       < 2e-16 ***
## relevel(city, "New York")StJohns        0.6260
## relevel(city, "New York")Tempe        2.92e-10 ***
## relevel(city, "New York")Toronto       < 2e-16 ***
## relevel(city, "New York")Tuscon       1.48e-07 ***
## relevel(city, "New York")Vancouver     < 2e-16 ***
## relevel(city, "New York")Victoria      < 2e-16 ***
## relevel(city, "New York")Washington    < 2e-16 ***
## ---
## Signif. codes:  0 '***' 0.001 '**' 0.01 '*' 0.05 '.' 0.1 ' ' 1
##
## Residual standard error: 2.682 on 5554 degrees of freedom
##   (85 observations deleted due to missingness)
## Multiple R-squared:  0.3474, Adjusted R-squared:  0.3445
## F-statistic: 123.2 on 24 and 5554 DF,  p-value: < 2.2e-16
```

```
confint(fe1)
```

```
##                                               2.5 %      97.5 %
## (Intercept)                            -2.920002841 -2.33733505
## avg_bikesc                              0.046854426  0.05509619
## relevel(city, "New York")Ann Arbor      1.394106203  3.24147988
## relevel(city, "New York")Austin         1.547184852  2.41295248
## relevel(city, "New York")Boston         0.044991946  0.88166791
## relevel(city, "New York")Calgary       -0.352982401  0.39442718
## relevel(city, "New York")Chicago        0.487593251  0.93316593
## relevel(city, "New York")Eugene         8.339997000 10.24565513
## relevel(city, "New York")Fort Collins   5.258024019  7.10933296
## relevel(city, "New York")Halifax        2.018624397  4.22351121
## relevel(city, "New York")Madison        4.299310312  5.77575347
## relevel(city, "New York")Minneapolis    2.049766737  3.06204641
## relevel(city, "New York")Moncton       -0.885026417  1.84313738
## relevel(city, "New York")Montreal       3.089295904  3.73896651
## relevel(city, "New York")Portland       4.948704886  5.87657666
## relevel(city, "New York")San Francisco  1.320517096  2.11488811
## relevel(city, "New York")Saskatoon      0.002191157  1.62655333
## relevel(city, "New York")Seattle        2.363006811  3.30709489
## relevel(city, "New York")StJohns       -0.813147982  1.35127673
## relevel(city, "New York")Tempe          1.939448556  3.68589879
## relevel(city, "New York")Toronto        0.953957217  1.46192704
## relevel(city, "New York")Tuscon         0.854231927  1.86863683
## relevel(city, "New York")Vancouver      2.290167940  3.30278063
## relevel(city, "New York")Victoria       9.085002890 11.64710163
## relevel(city, "New York")Washington     1.337605341  2.15843515
```

```
extractAIC(fe1)
```

```
## [1]    25.00 11034.58
```

Squared term for BikeScore

```
fe2<-lm(pc_bike_2012 ~ avg_bikesc + I(avg_bikesc^2) + relevel(city, "New York"), data=bikeScore)
summary(fe2)
```

```
##
## Call:
## lm(formula = pc_bike_2012 ~ avg_bikesc + I(avg_bikesc^2) + relevel(city,
##     "New York"), data = bikeScore)
##
## Residuals:
##     Min      1Q  Median      3Q     Max
## -10.423  -1.359  -0.336   0.521  32.375
##
## Coefficients:
##                                          Estimate Std. Error t value
## (Intercept)                            -0.2689855  0.4670145  -0.576
## avg_bikesc                             -0.0238379  0.0141965  -1.679
## I(avg_bikesc^2)                         0.0005494  0.0001031   5.328
## relevel(city, "New York")Ann Arbor      2.3641975  0.4700979   5.029
## relevel(city, "New York")Austin         1.7901080  0.2231390   8.022
## relevel(city, "New York")Boston         0.4375427  0.2129259   2.055
## relevel(city, "New York")Calgary        0.0940105  0.1906561   0.493
## relevel(city, "New York")Chicago        0.7664974  0.1138528   6.732
## relevel(city, "New York")Eugene         9.2460542  0.4849256  19.067
## relevel(city, "New York")Fort Collins   6.1784705  0.4710194  13.117
## relevel(city, "New York")Halifax        3.1918964  0.5611348   5.688
## relevel(city, "New York")Madison        5.0199752  0.3756578  13.363
## relevel(city, "New York")Minneapolis    2.5684446  0.2575595   9.972
## relevel(city, "New York")Moncton        0.3519493  0.6945216   0.507
## relevel(city, "New York")Montreal       3.3649473  0.1655497  20.326
## relevel(city, "New York")Portland       5.3731391  0.2361897  22.749
## relevel(city, "New York")San Francisco  1.6935963  0.2021581   8.378
## relevel(city, "New York")Saskatoon      0.8702855  0.4134108   2.105
## relevel(city, "New York")Seattle        2.7899300  0.2403484  11.608
## relevel(city, "New York")StJohns        0.0603552  0.5520741   0.109
## relevel(city, "New York")Tempe          2.8816174  0.4445281   6.482
## relevel(city, "New York")Toronto        1.2461660  0.1294389   9.627
## relevel(city, "New York")Tuscon         1.3336274  0.2581423   5.166
## relevel(city, "New York")Vancouver      2.8108033  0.2576475  10.909
## relevel(city, "New York")Victoria      10.3802871  0.6518663  15.924
## relevel(city, "New York")Washington     1.6934261  0.2090904   8.099
##                                        Pr(>|t|)
## (Intercept)                              0.5647
## avg_bikesc                               0.0932 .
## I(avg_bikesc^2)                        1.03e-07 ***
## relevel(city, "New York")Ann Arbor     5.08e-07 ***
## relevel(city, "New York")Austin        1.26e-15 ***
## relevel(city, "New York")Boston          0.0399 *
## relevel(city, "New York")Calgary         0.6220
## relevel(city, "New York")Chicago       1.84e-11 ***
## relevel(city, "New York")Eugene         < 2e-16 ***
## relevel(city, "New York")Fort Collins   < 2e-16 ***
## relevel(city, "New York")Halifax       1.35e-08 ***
## relevel(city, "New York")Madison        < 2e-16 ***
## relevel(city, "New York")Minneapolis    < 2e-16 ***
## relevel(city, "New York")Moncton         0.6123
## relevel(city, "New York")Montreal       < 2e-16 ***
## relevel(city, "New York")Portland       < 2e-16 ***
## relevel(city, "New York")San Francisco  < 2e-16 ***
## relevel(city, "New York")Saskatoon       0.0353 *
## relevel(city, "New York")Seattle        < 2e-16 ***
## relevel(city, "New York")StJohns         0.9129
## relevel(city, "New York")Tempe         9.81e-11 ***
## relevel(city, "New York")Toronto        < 2e-16 ***
## relevel(city, "New York")Tuscon        2.47e-07 ***
## relevel(city, "New York")Vancouver      < 2e-16 ***
## relevel(city, "New York")Victoria       < 2e-16 ***
## relevel(city, "New York")Washington    6.75e-16 ***
## ---
## Signif. codes:  0 '***' 0.001 '**' 0.01 '*' 0.05 '.' 0.1 ' ' 1
##
## Residual standard error: 2.676 on 5553 degrees of freedom
##   (85 observations deleted due to missingness)
## Multiple R-squared:  0.3507, Adjusted R-squared:  0.3477
## F-statistic:   120 on 25 and 5553 DF,  p-value: < 2.2e-16
```

```
confint(fe2)
```

```
##                                                   2.5 %       97.5 %
## (Intercept)                             -1.1845166131 6.465456e-01
## avg_bikesc                              -0.0516685911 3.992864e-03
## I(avg_bikesc^2)                          0.0003472651 7.515407e-04
## relevel(city, "New York")Ann Arbor       1.4426217296 3.285773e+00
## relevel(city, "New York")Austin          1.3526682962 2.227548e+00
## relevel(city, "New York")Boston          0.0201246218 8.549608e-01
## relevel(city, "New York")Calgary        -0.2797501542 4.677711e-01
## relevel(city, "New York")Chicago         0.5433013334 9.896934e-01
## relevel(city, "New York")Eugene          8.2954101490 1.019670e+01
## relevel(city, "New York")Fort Collins    5.2550880792 7.101853e+00
## relevel(city, "New York")Halifax         2.0918526725 4.291940e+00
## relevel(city, "New York")Madison         4.2835388897 5.756412e+00
## relevel(city, "New York")Minneapolis     2.0635271241 3.073362e+00
## relevel(city, "New York")Moncton        -1.0095848068 1.713483e+00
## relevel(city, "New York")Montreal        3.0404051335 3.689489e+00
## relevel(city, "New York")Portland        4.9101147995 5.836163e+00
## relevel(city, "New York")San Francisco  1.2972873357 2.089905e+00
## relevel(city, "New York")Saskatoon       0.0598385796 1.680732e+00
## relevel(city, "New York")Seattle         2.3187531313 3.261107e+00
## relevel(city, "New York")StJohns        -1.0219260291 1.142636e+00
## relevel(city, "New York")Tempe           2.0101683222 3.753067e+00
## relevel(city, "New York")Toronto         0.9924150429 1.499917e+00
## relevel(city, "New York")Tuscon          0.8275675972 1.839687e+00
## relevel(city, "New York")Vancouver       2.3057133280 3.315893e+00
## relevel(city, "New York")Victoria        9.1023741753 1.165820e+01
## relevel(city, "New York")Washington      1.2835270562 2.103325e+00
```

```
extractAIC(fe2)
```

```
## [1]    26.00 11008.12
```

Plot for Fixed Effects Model

```
library(splines)
bikeScore$fe1fit <- NA
bikeScore$fe1fit[!is.na(bikeScore$pc_bike_2012)] <- fitted(fe1)
bikeScore$pre.fe1 <- exp(bikeScore$fe1fit)/(1 + exp(bikeScore$fe1fit))

bikeScore$fe2fit <- NA
bikeScore$fe2fit[!is.na(bikeScore$pc_bike_2012)] <- fitted(fe2)
```

Categorized Bike Score - Fixed Effect

```
library(car)
```

```
##
## Attaching package: 'car'
##
## The following object is masked from 'package:psych':
##
##     logit
```

```
bikeScore$avg_bikesc_cat <- recode(bikeScore$avg_bikesc,
                      "0:25='1 - 0 to 25';
                      25.001:50='2 - 25+ to 50';
                      50.001:75='3 - 50+ to 75';
                      75.001:90='4 - 75+ to 90';
                      90.001:100='5 - 90+ to 100';",
                as.factor.result=TRUE)
library(xtable)
bikeScorecat <- table(bikeScore$avg_bikesc_cat)
bikeScorecat <- data.frame(bikeScorecat)
colnames(bikeScorecat) <- c("Bike Score Category", "n")
print(xtable(bikeScorecat), type = "html", )
```

	Bike Score Category	n
1	1 - 0 to 25	29
2	2 - 25+ to 50	1267
3	3 - 50+ to 75	2357

4	4 - 75+ to 90	1202
5	5 - 90+ to 100	807

Categorization of Bikescore linear regression on % cycling

```
library(car)
m1_cat<-lm(pc_bike_2012 ~ factor(avg_bikesc_cat), data=bikeScore)
summary(m1_cat)
```

```
##
## Call:
## lm(formula = pc_bike_2012 ~ factor(avg_bikesc_cat), data = bikeScore)
##
## Residuals:
##     Min      1Q  Median      3Q     Max
## -4.202 -1.557 -0.529  0.511 32.293
##
## Coefficients:
##                                      Estimate Std. Error t value Pr(>|t|)
## (Intercept)                            0.7233     0.5971   1.211   0.2258
## factor(avg_bikesc_cat)2 - 25+ to 50   -0.1941     0.6036  -0.322   0.7478
## factor(avg_bikesc_cat)3 - 50+ to 75    0.8336     0.6006   1.388   0.1652
## factor(avg_bikesc_cat)4 - 75+ to 90    1.9565     0.6038   3.240   0.0012
## factor(avg_bikesc_cat)5 - 90+ to 100   3.4785     0.6071   5.730 1.06e-08
##
## (Intercept)
## factor(avg_bikesc_cat)2 - 25+ to 50
## factor(avg_bikesc_cat)3 - 50+ to 75
## factor(avg_bikesc_cat)4 - 75+ to 90  **
## factor(avg_bikesc_cat)5 - 90+ to 100 ***
## ---
## Signif. codes:  0 '***' 0.001 '**' 0.01 '*' 0.05 '.' 0.1 ' ' 1
##
## Residual standard error: 3.103 on 5574 degrees of freedom
##   (85 observations deleted due to missingness)
## Multiple R-squared:  0.1237, Adjusted R-squared:  0.123
## F-statistic: 196.7 on 4 and 5574 DF,  p-value: < 2.2e-16
```

```
confint(m1_cat)
```

```
##                                           2.5 %   97.5 %
## (Intercept)                          -0.4472253 1.893892
## factor(avg_bikesc_cat)2 - 25+ to 50  -1.3773797 0.989252
## factor(avg_bikesc_cat)3 - 50+ to 75  -0.3437192 2.010912
## factor(avg_bikesc_cat)4 - 75+ to 90   0.7727407 3.140290
## factor(avg_bikesc_cat)5 - 90+ to 100  2.2883593 4.668704
```

Categorization of Bikescore linear regression on % cycling with City level fixed effects

```
library(car)
m2_cat<-lm(pc_bike_2012 ~ factor(avg_bikesc_cat) + relevel(city, "New York"), data=bikeScore)
summary(m2_cat)
```

```
##
## Call:
## lm(formula = pc_bike_2012 ~ factor(avg_bikesc_cat) + relevel(city,
##     "New York"), data = bikeScore)
##
## Residuals:
##     Min      1Q  Median      3Q     Max
## -10.611  -1.226  -0.465   0.520  32.115
##
## Coefficients:
##                                         Estimate Std. Error t value
## (Intercept)                              -1.3565     0.5339  -2.541
## factor(avg_bikesc_cat)2 - 25+ to 50      1.1054     0.5352   2.065
## factor(avg_bikesc_cat)3 - 50+ to 75      1.8218     0.5338   3.413
## factor(avg_bikesc_cat)4 - 75+ to 90      2.5822     0.5378   4.801
## factor(avg_bikesc_cat)5 - 90+ to 100     3.9835     0.5409   7.365
## relevel(city, "New York")Ann Arbor       2.4720     0.4722   5.235
## relevel(city, "New York")Austin          1.7507     0.2217   7.898
## relevel(city, "New York")Boston          0.4806     0.2139   2.247
## relevel(city, "New York")Calgary         0.2504     0.1930   1.297
## relevel(city, "New York")Chicago         0.7449     0.1145   6.508
## relevel(city, "New York")Eugene          9.3854     0.4874  19.255
## relevel(city, "New York")Fort Collins    6.2612     0.4734  13.226
## relevel(city, "New York")Halifax         3.3057     0.5639   5.862
## relevel(city, "New York")Madison         5.0952     0.3774  13.502
## relevel(city, "New York")Minneapolis     2.6632     0.2587  10.293
## relevel(city, "New York")Moncton         0.2742     0.6973   0.393
## relevel(city, "New York")Montreal        3.4460     0.1659  20.768
## relevel(city, "New York")Portland        5.4383     0.2385  22.800
## relevel(city, "New York")San Francisco   1.7754     0.2039   8.709
## relevel(city, "New York")Saskatoon       1.0102     0.4158   2.430
## relevel(city, "New York")Seattle         2.7834     0.2414  11.532
## relevel(city, "New York")StJohns         0.1070     0.5590   0.191
## relevel(city, "New York")Tempe           3.0611     0.4467   6.852
## relevel(city, "New York")Toronto         1.2693     0.1303   9.743
## relevel(city, "New York")Tuscon          1.3829     0.2593   5.334
## relevel(city, "New York")Vancouver       2.8691     0.2588  11.085
## relevel(city, "New York")Victoria       10.2831     0.6549  15.702
## relevel(city, "New York")Washington      1.6675     0.2099   7.945
##                                         Pr(>|t|)
## (Intercept)                             0.011093 *
## factor(avg_bikesc_cat)2 - 25+ to 50     0.038934 *
## factor(avg_bikesc_cat)3 - 50+ to 75     0.000647 ***
## factor(avg_bikesc_cat)4 - 75+ to 90     1.62e-06 ***
## factor(avg_bikesc_cat)5 - 90+ to 100    2.03e-13 ***
## relevel(city, "New York")Ann Arbor      1.71e-07 ***
## relevel(city, "New York")Austin         3.39e-15 ***
## relevel(city, "New York")Boston         0.024688 *
## relevel(city, "New York")Calgary        0.194572
## relevel(city, "New York")Chicago        8.28e-11 ***
## relevel(city, "New York")Eugene          < 2e-16 ***
## relevel(city, "New York")Fort Collins    < 2e-16 ***
## relevel(city, "New York")Halifax        4.83e-09 ***
## relevel(city, "New York")Madison         < 2e-16 ***
## relevel(city, "New York")Minneapolis     < 2e-16 ***
## relevel(city, "New York")Moncton        0.694126
## relevel(city, "New York")Montreal        < 2e-16 ***
## relevel(city, "New York")Portland        < 2e-16 ***
## relevel(city, "New York")San Francisco   < 2e-16 ***
## relevel(city, "New York")Saskatoon      0.015151 *
## relevel(city, "New York")Seattle         < 2e-16 ***
## relevel(city, "New York")StJohns        0.848247
## relevel(city, "New York")Tempe          8.07e-12 ***
## relevel(city, "New York")Toronto         < 2e-16 ***
## relevel(city, "New York")Tuscon         9.99e-08 ***
## relevel(city, "New York")Vancouver       < 2e-16 ***
## relevel(city, "New York")Victoria        < 2e-16 ***
## relevel(city, "New York")Washington     2.33e-15 ***
## ---
## Signif. codes:  0 '***' 0.001 '**' 0.01 '*' 0.05 '.' 0.1 ' ' 1
##
## Residual standard error: 2.688 on 5551 degrees of freedom
##   (85 observations deleted due to missingness)
## Multiple R-squared:  0.3452, Adjusted R-squared:  0.342
## F-statistic: 108.4 on 27 and 5551 DF,  p-value: < 2.2e-16
```

```
confint(m2_cat)
```

```
##                                               2.5 %     97.5 %
## (Intercept)                             -2.40322401 -0.3097937
## factor(avg_bikesc_cat)2 - 25+ to 50      0.05619052  2.1546894
## factor(avg_bikesc_cat)3 - 50+ to 75      0.77541655  2.8681954
## factor(avg_bikesc_cat)4 - 75+ to 90      1.52790664  3.6365570
## factor(avg_bikesc_cat)5 - 90+ to 100     2.92319431  5.0437987
## relevel(city, "New York")Ann Arbor      1.54622863  3.3977900
## relevel(city, "New York")Austin         1.31614158  2.1852072
## relevel(city, "New York")Boston         0.06127128  0.8998556
## relevel(city, "New York")Calgary       -0.12798950  0.6288297
## relevel(city, "New York")Chicago        0.52050739  0.9692687
## relevel(city, "New York")Eugene         8.42984017 10.3409125
## relevel(city, "New York")Fort Collins   5.33314732  7.1892639
## relevel(city, "New York")Halifax        2.20024597  4.4110778
## relevel(city, "New York")Madison        4.35546160  5.8349881
## relevel(city, "New York")Minneapolis    2.15599307  3.1704194
## relevel(city, "New York")Moncton       -1.09269476  1.6411416
## relevel(city, "New York")Montreal       3.12070679  3.7712755
## relevel(city, "New York")Portland       4.97066025  5.9058652
## relevel(city, "New York")San Francisco  1.37574873  2.1750490
## relevel(city, "New York")Saskatoon      0.19507150  1.8254213
## relevel(city, "New York")Seattle        2.31022918  3.2565669
## relevel(city, "New York")StJohns       -0.98890389  1.2028528
## relevel(city, "New York")Tempe          2.18531105  3.9368974
## relevel(city, "New York")Toronto        1.01393418  1.5247147
## relevel(city, "New York")Tuscon         0.87462017  1.8910993
## relevel(city, "New York")Vancouver      2.36167773  3.3764327
## relevel(city, "New York")Victoria       8.99922749 11.5669018
## relevel(city, "New York")Washington     1.25603944  2.0789100
```

```
extractAIC(m2_cat)
```

```
## [1]    28.00 11058.98
```

Bikescore - Random slopes model

A. Multilevel with Random intercept for city

```
library(nlme)
```

```
## Warning: package 'nlme' was built under R version 3.1.2
```

```
mlmA<-lme(pc_bike_2012 ~ 1, random = ~ 1 | city, data=bikeScore, na.action=na.exclude)
summary(mlmA)
```

```
## Linear mixed-effects model fit by REML
##  Data: bikeScore
##        AIC      BIC    logLik
##   27523.01 27542.89 -13758.51
##
## Random effects:
##  Formula: ~1 | city
##         (Intercept) Residual
## StdDev:      2.8967 2.820438
##
## Fixed effects: pc_bike_2012 ~ 1
##                Value Std.Error   DF  t-value p-value
## (Intercept) 3.693613 0.5964103 5556 6.193074       0
##
## Standardized Within-Group Residuals:
##        Min         Q1        Med         Q3        Max
## -3.6984920 -0.4152753 -0.2438410  0.2125460 11.2964118
##
## Number of Observations: 5580
## Number of Groups: 24
```

B. MLM with random intercept for city and country

```
mlmB<-lme(pc_bike_2012 ~ 1, random= ~ 1 | Country/city, data=bikeScore, na.action=na.exclude)
summary(mlmB)
```

```
## Linear mixed-effects model fit by REML
##  Data: bikeScore
##        AIC      BIC    logLik
##   27525.01 27551.52 -13758.51
##
## Random effects:
##  Formula: ~1 | Country
##         (Intercept)
## StdDev: 0.002657606
##
##  Formula: ~1 | city %in% Country
##         (Intercept) Residual
## StdDev:      2.8967 2.820438
##
## Fixed effects: pc_bike_2012 ~ 1
##                Value Std.Error   DF  t-value p-value
## (Intercept) 3.693612 0.5964136 5556 6.193038       0
##
## Standardized Within-Group Residuals:
##        Min         Q1        Med         Q3        Max
## -3.6984920 -0.4152753 -0.2438410  0.2125460 11.2964118
##
## Number of Observations: 5580
## Number of Groups:
##           Country city %in% Country
##                 2                24
```

Compare model A and B

```
anova(mlmA, mlmB)
```

```
##      Model df      AIC      BIC    logLik   Test      L.Ratio p-value
## mlmA     1  3 27523.01 27542.89 -13758.51
## mlmB     2  4 27525.01 27551.52 -13758.51 1 vs 2 6.547787e-06   0.998
```

Adding Country does not improve the model

C. MLM with BikeScore and random intercept for city

```
mlmC<-lme(pc_bike_2012 ~ avg_bikesc, random= ~ 1 | city, data=bikeScore, na.action=na.exclude)
summary(mlmC)
```

```
## Linear mixed-effects model fit by REML
##  Data: bikeScore
##        AIC      BIC    logLik
##   26968.59 26995.09 -13480.29
##
## Random effects:
##  Formula: ~1 | city
##         (Intercept) Residual
## StdDev:    2.666911 2.682466
##
## Fixed effects: pc_bike_2012 ~ avg_bikesc
##                  Value Std.Error   DF  t-value p-value
## (Intercept) 0.13107760 0.5685689 5554  0.23054  0.8177
## avg_bikesc  0.05111627 0.0021006 5554 24.33417  0.0000
##  Correlation:
##            (Intr)
## avg_bikesc -0.257
##
## Standardized Within-Group Residuals:
##        Min         Q1        Med         Q3        Max
## -3.8781236 -0.5206655 -0.1372669  0.2228102 12.0303755
##
## Number of Observations: 5579
## Number of Groups: 24
```

D. MLM with BikeScore and random intercept for city and country

```
mlmD<-lme(pc_bike_2012 ~ avg_bikesc, random= ~ 1 | Country/city, data=bikeScore, na.action=na.exclude, method="ML")
summary(mlmD)
```

```
## Linear mixed-effects model fit by maximum likelihood
##  Data: bikeScore
##        AIC      BIC    logLik
##   26960.71 26993.85 -13475.36
##
## Random effects:
##  Formula: ~1 | Country
##         (Intercept)
## StdDev: 0.001445925
##
##  Formula: ~1 | city %in% Country
##         (Intercept) Residual
## StdDev:     2.60773 2.682233
##
## Fixed effects: pc_bike_2012 ~ avg_bikesc
##                  Value Std.Error   DF   t-value p-value
## (Intercept) 0.12981791 0.5571058 5554  0.233022  0.8158
## avg_bikesc  0.05112243 0.0021007 5554 24.335654  0.0000
##  Correlation:
##            (Intr)
## avg_bikesc -0.263
##
## Standardized Within-Group Residuals:
##        Min         Q1        Med         Q3        Max
## -3.8750520 -0.5203218 -0.1372445  0.2227290 12.0313899
##
## Number of Observations: 5579
## Number of Groups:
##           Country city %in% Country
##                 2                24
```

E. MLM with BikeScore and random intercept and slope for city

```
mlmE<-lme(pc_bike_2012 ~ avg_bikesc, random= ~ avg_bikesc | city, data=bikeScore, na.action=na.exclude)
summary(mlmE)
```

```
## Linear mixed-effects model fit by REML
##  Data: bikeScore
##        AIC      BIC    logLik
##   26564.46 26604.22 -13276.23
##
## Random effects:
##  Formula: ~avg_bikesc | city
##  Structure: General positive-definite, Log-Cholesky parametrization
##             StdDev     Corr
## (Intercept) 5.42388737 (Intr)
## avg_bikesc  0.07282489 -0.879
## Residual    2.56972505
##
## Fixed effects: pc_bike_2012 ~ avg_bikesc
##                 Value Std.Error   DF   t-value p-value
## (Intercept) -2.004097 1.1565546 5554 -1.732816  0.0832
## avg_bikesc   0.078464 0.0155662 5554  5.040653  0.0000
##  Correlation:
##            (Intr)
## avg_bikesc -0.886
##
## Standardized Within-Group Residuals:
##        Min         Q1        Med         Q3        Max
## -4.0402653 -0.4365488 -0.1655306  0.2049288 12.5774656
##
## Number of Observations: 5579
## Number of Groups: 24
```

F. MLM with BikeScore and random intercept and slope for city and country

```
mlmF<-lme(pc_bike_2012 ~ avg_bikesc, random= ~ avg_bikesc | Country/city, data=bikeScore, na.action=na.exclude, method="ML")
summary(mlmF)
```

```
## Linear mixed-effects model fit by maximum likelihood
##  Data: bikeScore 
##        AIC      BIC    logLik
##   26564.51 26624.15 -13273.26
## 
## Random effects:
##  Formula: ~avg_bikesc | Country
##  Structure: General positive-definite, Log-Cholesky parametrization
##             StdDev       Corr  
## (Intercept) 1.256585e-10 (Intr)
## avg_bikesc  2.130859e-05 -0.007
## 
##  Formula: ~avg_bikesc | city %in% Country
##  Structure: General positive-definite, Log-Cholesky parametrization
##             StdDev     Corr  
## (Intercept) 5.24683274 (Intr)
## avg_bikesc  0.07061468 -0.876
## Residual    2.56982654       
## 
## Fixed effects: pc_bike_2012 ~ avg_bikesc 
##                  Value Std.Error   DF   t-value p-value
## (Intercept) -2.0027931 1.1218521 5554 -1.785256  0.0743
## avg_bikesc   0.0784734 0.0151337 5554  5.185345  0.0000
##  Correlation: 
##            (Intr)
## avg_bikesc -0.884
## 
## Standardized Within-Group Residuals:
##        Min         Q1        Med         Q3        Max 
## -4.0370229 -0.4364744 -0.1655157  0.2057495 12.5769546 
## 
## Number of Observations: 5579
## Number of Groups: 
##           Country city %in% Country 
##                 2                24
```

Compare model C and E

```
anova(mlmC, mlmE)
```

```
##      Model df      AIC      BIC    logLik   Test  L.Ratio p-value
## mlmC     1  4 26968.59 26995.09 -13480.29                        
## mlmE     2  6 26564.46 26604.22 -13276.23 1 vs 2 408.1271  <.0001
```

Model E fits better than model C

Compare model D and F

```
anova(mlmD, mlmF)
```

```
##      Model df      AIC      BIC    logLik   Test  L.Ratio p-value
## mlmD     1  5 26960.71 26993.85 -13475.36                        
## mlmF     2  9 26564.51 26624.15 -13273.26 1 vs 2 404.1989  <.0001
```

Model F fits better than model D

```
mlm5<-lme(pc_bike_2012 ~ avg_bikesc + citypop + citypopdenssqkm + AverageTempJan + AverageTempJul + TotalWetDays, random= ~av
g_bikesc|city, data=bikeScore, na.action=na.exclude, control=list(opt="optim"))
summary(mlm5)
```

```
## Linear mixed-effects model fit by REML
##  Data: bikeScore
##        AIC      BIC    logLik
##   26619.99 26692.87 -13298.99
##
## Random effects:
##  Formula: ~avg_bikesc | city
##  Structure: General positive-definite, Log-Cholesky parametrization
##             StdDev     Corr
## (Intercept) 5.17379945 (Intr)
## avg_bikesc  0.07236551 -0.887
## Residual    2.56979156
##
## Fixed effects: pc_bike_2012 ~ avg_bikesc + citypop + citypopdenssqkm + AverageTempJan +      AverageTempJul + TotalWetDays
##                      Value Std.Error   DF   t-value p-value
## (Intercept)     -0.9252716  4.861175 5554 -0.190339  0.8491
## avg_bikesc       0.0790219  0.015477 5554  5.105874  0.0000
## citypop         -0.0000004  0.000001   18 -0.777592  0.4469
## citypopdenssqkm -0.0000203  0.000370   18 -0.055028  0.9567
## AverageTempJan   0.1527161  0.084450   18  1.808371  0.0873
## AverageTempJul  -0.1605721  0.157023   18 -1.022602  0.3200
## TotalWetDays     0.0258513  0.018239   18  1.417376  0.1735
##  Correlation:
##                 (Intr) avg_bk citypp ctyppd AvrgTmpJn AvrgTmpJl
## avg_bikesc      -0.212
## citypop          0.352  0.011
## citypopdenssqkm -0.346 -0.009 -0.810
## AverageTempJan   0.352  0.005  0.385 -0.433
## AverageTempJul  -0.893  0.000 -0.381  0.338 -0.477
## TotalWetDays    -0.670  0.021 -0.027 -0.065  0.117     0.377
##
## Standardized Within-Group Residuals:
##        Min         Q1        Med         Q3        Max
## -4.0525866 -0.4364587 -0.1659013  0.2060433 12.5776772
##
## Number of Observations: 5579
## Number of Groups: 24
```

```
intervals(mlm5)
```

```
## Approximate 95% confidence intervals
##
##  Fixed effects:
##                         lower          est.        upper
## (Intercept)     -1.045508e+01 -9.252716e-01 8.604534e+00
## avg_bikesc       4.868161e-02  7.902194e-02 1.093623e-01
## citypop         -1.480106e-06 -3.998307e-07 6.804444e-07
## citypopdenssqkm -7.968772e-04 -2.033937e-05 7.561985e-04
## AverageTempJan  -2.470584e-02  1.527161e-01 3.301380e-01
## AverageTempJul  -4.904654e-01 -1.605721e-01 1.693212e-01
## TotalWetDays    -1.246708e-02  2.585130e-02 6.416969e-02
## attr(,"label")
## [1] "Fixed effects:"
##
##  Random Effects:
##   Level: city
##                                   lower        est.      upper
## sd((Intercept))              3.49077319  5.17379945  7.6682727
## sd(avg_bikesc)               0.05089887  0.07236551  0.1028857
## cor((Intercept),avg_bikesc) -0.95778465 -0.88737569 -0.7165376
##
##  Within-group standard error:
##    lower     est.    upper
## 2.522307 2.569792 2.618170
```

```
bikeScore$mlm5fit <- fitted(mlm5)
```

```
fig_mlm5 <- ggplot(bikeScore, aes(x=avg_bikesc, y=mlm5fit, colour=factor(city))) +
          geom_point() +
          stat_smooth(method = "lm", formula = y ~ x, color="black", se = TRUE) +
          theme(axis.line = element_line(colour = "black"),
          panel.grid.major = element_blank(),
          panel.grid.minor = element_blank(),
          panel.border = element_blank(),
          panel.background = element_blank(),
          axis.title=element_text(size=16,face="bold"),
          axis.text=element_text(size=14))
plot(fig_mlm5)
```

```
## Warning: Removed 85 rows containing missing values (stat_smooth).
```

```
## Warning: Removed 85 rows containing missing values (geom_point).
```

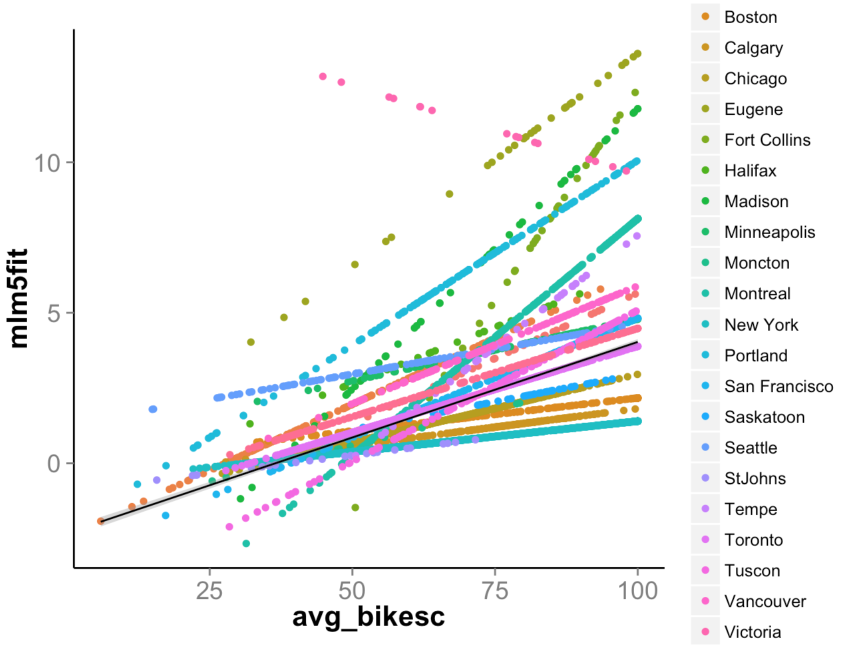


```
ggsave(fig_mlm5, file="/Users/DogLeg/Dropbox/Manuscripts/2015 Bikescore/Random Slopes Regression.pdf", width = 13, height = 10,
dpi = 600)
```

```
## Warning: Removed 85 rows containing missing values (stat_smooth).
```

```
## Warning: Removed 85 rows containing missing values (geom_point).
```

ModeShare Tranforms

Histogram of modeshare data

```
library(stats)

Histo_modeshare <- ggplot(bikeScore, aes(pc_bike_2012)) +
              geom_histogram(colour = "black", fill = "white", binwidth = 0.5) +
          xlab('Mode Share') +
          ylab('Percent Biking to Work')
plot(Histo_modeshare)
```

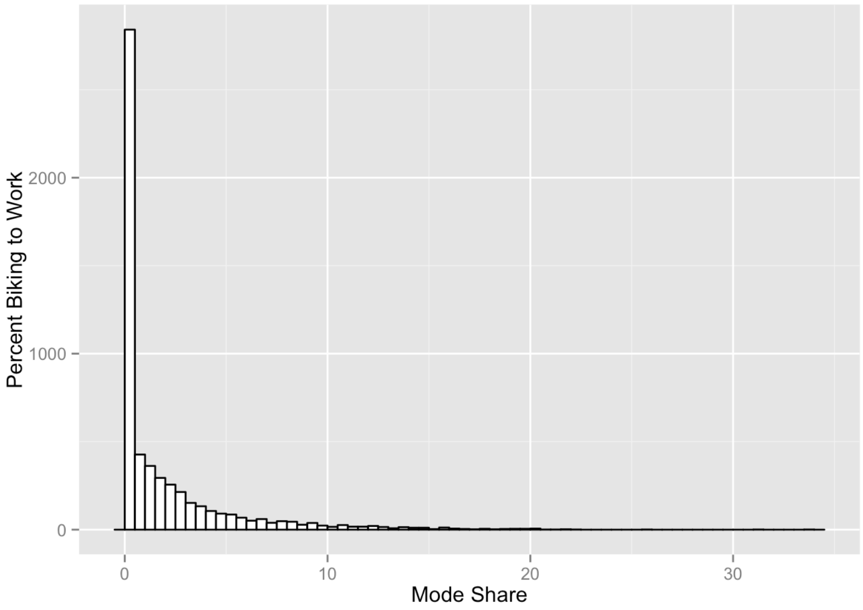


```
ggsave(Histo_modeshare, file="/Users/DogLeg/Dropbox/Manuscripts/2015 Bikescore/ModeShare Histogram.pdf", width = 13, height = 1
0, dpi = 600)
```

Logit Tranform

```
### First Add 1 to all values. This maps 0 to 0 when transformed.

bikeScore$t_pc_bike_2012_1 <- bikeScore$pc_bike_2012+1
bikeScore$t_pc_bike_2012 <- log(bikeScore$t_pc_bike_2012_1)
psych::describe(bikeScore$t_pc_bike_2012)
```

```
##   vars    n mean   sd median trimmed  mad min  max range skew kurtosis
## 1    1 5580 0.69 0.81   0.37    0.56 0.55   0 3.55  3.55 0.92    -0.25
##     se
## 1 0.01
```

```
modeshareLogit <- ggplot(bikeScore, aes(t_pc_bike_2012)) +
              geom_histogram(colour = "black", fill = "white", binwidth = 0.5) +
          xlab('Logit Transformed Mode Share') +
          ylab('Percent Biking to Work')
plot(modeshareLogit)
```

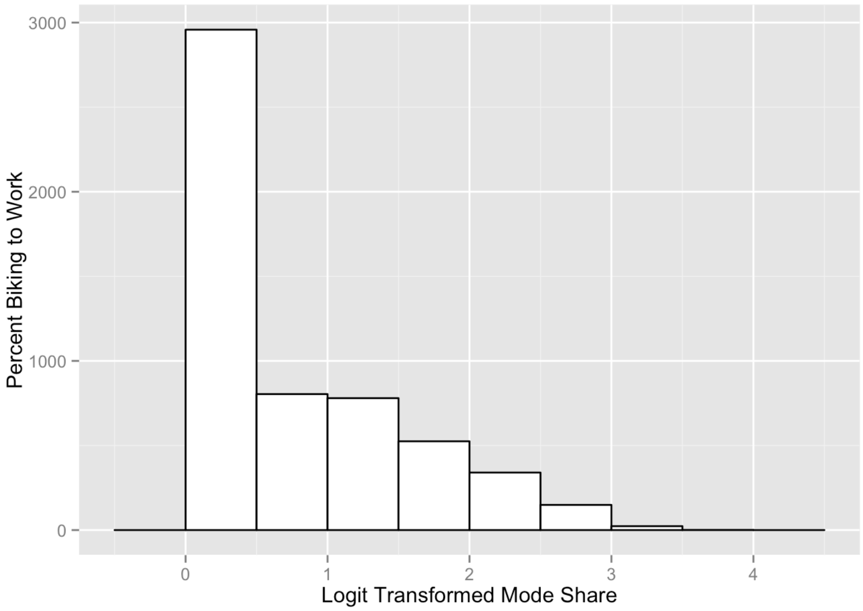

The logit transform helps somewhat but the data is still highly skewed. This looks like count data but it is not integer data. It would be temping to do a Poisson or negative binomial model but these models require integer data.

Converting to % bike to integer

```
bikeScore$int_pc_bike_2012 <- as.integer(bikeScore$t_pc_bike_2012)
modeshareLogitInt <- ggplot(bikeScore, aes(int_pc_bike_2012)) +
              geom_histogram(colour = "black", fill = "white", binwidth = 0.5) +
          xlab('Mode Share as integer') +
          ylab('Percent Biking to Work')
plot(modeshareLogitInt)
```

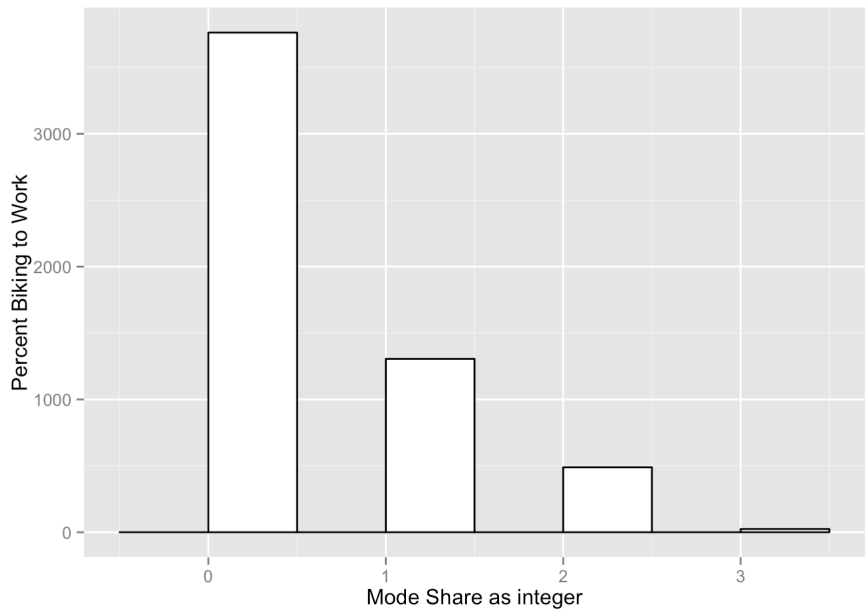

This does not help the problem

Box-Cox Tranform

```
library(geoR)
```

```
## Warning: package 'geoR' was built under R version 3.1.3
```

```
## --------------------------------------------------------------
##  Analysis of Geostatistical Data
##  For an Introduction to geoR go to http://www.leg.ufpr.br/geoR
##  geoR version 1.7-5.1 (built on 2015-04-15) is now loaded
## --------------------------------------------------------------
```

```
bikeScore$bc_pc_bike_2012 <- bcPower(bikeScore$pc_bike_2012+0.1, 0)

modeshareBCTransform <- ggplot(bikeScore, aes(bc_pc_bike_2012)) +
              geom_histogram(colour = "black", fill = "white", binwidth = 0.5) +
          xlab('Box-Cox Tranformed Mode Share') +
          ylab('Percent Biking to Work')
plot(modeshareBCTransform)
```

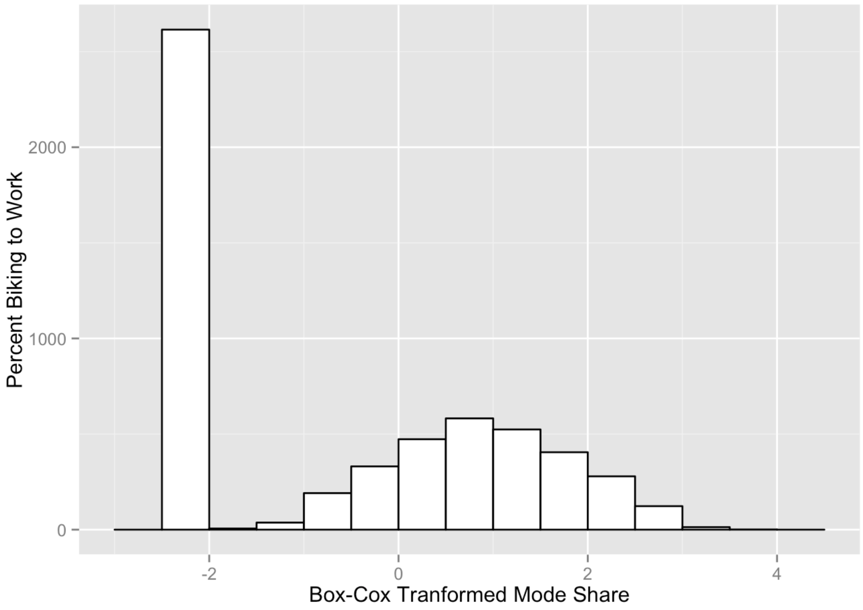

This type of transform requires a mixture model which models 0 and the remaining distribution separately. We believe this is out of scope for this paper.

Bikescore Components - Random Intercept Model

```
biv_avg_stret <- lm(pc_bike_2012 ~ avg_stret, data=bikeScore)
summary(biv_avg_stret)
```

```
##
## Call:
## lm(formula = pc_bike_2012 ~ avg_stret, data = bikeScore)
##
## Residuals:
##    Min     1Q Median     3Q    Max
## -2.208 -2.108 -1.241  0.573 33.279
##
## Coefficients:
##             Estimate Std. Error t value Pr(>|t|)
## (Intercept) 0.551853   0.161297   3.421 0.000628 ***
## avg_stret   0.016563   0.001846   8.972  < 2e-16 ***
## ---
## Signif. codes:  0 '***' 0.001 '**' 0.01 '*' 0.05 '.' 0.1 ' ' 1
##
## Residual standard error: 3.29 on 5577 degrees of freedom
##   (85 observations deleted due to missingness)
## Multiple R-squared:  0.01423,    Adjusted R-squared:  0.01405
## F-statistic:  80.5 on 1 and 5577 DF,  p-value: < 2.2e-16
```

```
confint(biv_avg_stret)
```

```
##                  2.5 %     97.5 %
## (Intercept) 0.23564759 0.86805916
## avg_stret   0.01294412 0.02018188
```

```
biv_avg_roughn <- lm(pc_bike_2012 ~ avg_roughn, data=bikeScore)
summary(biv_avg_roughn)
```

```
##
## Call:
## lm(formula = pc_bike_2012 ~ avg_roughn, data = bikeScore)
##
## Residuals:
##    Min     1Q Median     3Q    Max
## -2.910 -1.860 -1.499  0.553 31.990
##
## Coefficients:
##              Estimate Std. Error t value Pr(>|t|)
## (Intercept)  2.910279   0.250535  11.616  < 2e-16 ***
## avg_roughn  -0.010507   0.002681  -3.918 9.02e-05 ***
## ---
## Signif. codes:  0 '***' 0.001 '**' 0.01 '*' 0.05 '.' 0.1 ' ' 1
##
## Residual standard error: 3.309 on 5577 degrees of freedom
##   (85 observations deleted due to missingness)
## Multiple R-squared:  0.002745,   Adjusted R-squared:  0.002567
## F-statistic: 15.35 on 1 and 5577 DF,  p-value: 9.024e-05
```

```
confint(biv_avg_roughn)
```

```
##                   2.5 %       97.5 %
## (Intercept)  2.41913311  3.401424713
## avg_roughn  -0.01576361 -0.005250138
```

```
biv_avg_bikela <- lm(pc_bike_2012 ~ avg_bikela, data=bikeScore)
summary(biv_avg_bikela)
```

```
##
## Call:
## lm(formula = pc_bike_2012 ~ avg_bikela, data = bikeScore)
##
## Residuals:
##     Min      1Q  Median      3Q     Max
## -3.6157 -1.6650 -0.6182  0.4753 31.2537
##
## Coefficients:
##             Estimate Std. Error t value Pr(>|t|)
## (Intercept) 0.484847   0.068747   7.053 1.97e-12 ***
## avg_bikela  0.031309   0.001172  26.724  < 2e-16 ***
## ---
## Signif. codes:  0 '***' 0.001 '**' 0.01 '*' 0.05 '.' 0.1 ' ' 1
##
## Residual standard error: 3.12 on 5577 degrees of freedom
##   (85 observations deleted due to missingness)
## Multiple R-squared:  0.1135, Adjusted R-squared:  0.1134
## F-statistic: 714.1 on 1 and 5577 DF,  p-value: < 2.2e-16
```

```
confint(biv_avg_bikela)
```

```
##                  2.5 %     97.5 %
## (Intercept) 0.35007552 0.61961815
## avg_bikela  0.02901201 0.03360551
```

```
cs_mlm<-lme(pc_bike_2012~avg_stret + avg_bikela + avg_roughn + citypop + citypopdenssqkm + AverageTempJul, random = ~1|city, da
ta=bikeScore, na.action=na.exclude, control=list(opt="optim"))
summary(cs_mlm)
```

```
## Linear mixed-effects model fit by REML
##  Data: bikeScore
##        AIC      BIC    logLik
##   26873.48 26933.11 -13427.74
##
## Random effects:
##  Formula: ~1 | city
##         (Intercept) Residual
## StdDev:    2.616876 2.642804
##
## Fixed effects: pc_bike_2012 ~ avg_stret + avg_bikela + avg_roughn + citypop +      citypopdenssqkm + AverageTempJul
##                     Value Std.Error   DF   t-value p-value
## (Intercept)      1.2029211 3.0104526 5552  0.399581  0.6895
## avg_stret        0.0355773 0.0018444 5552 19.289138  0.0000
## avg_bikela       0.0189886 0.0011594 5552 16.378360  0.0000
## avg_roughn       0.0124960 0.0027667 5552  4.516619  0.0000
## citypop         -0.0000006 0.0000005   20 -1.238476  0.2299
## citypopdenssqkm  0.0000135 0.0003631   20  0.037078  0.9708
## AverageTempJul  -0.0800603 0.1304042   20 -0.613939  0.5462
##  Correlation:
##                 (Intr) avg_st avg_bk avg_rg citypp ctyppd
## avg_stret       -0.020
## avg_bikela      -0.026 -0.138
## avg_roughn      -0.060 -0.107  0.039
## citypop          0.317  0.019  0.011 -0.020
## citypopdenssqkm -0.398 -0.044  0.000  0.020 -0.777
## AverageTempJul  -0.950  0.000  0.004 -0.021 -0.234  0.198
##
## Standardized Within-Group Residuals:
##        Min         Q1        Med         Q3        Max
## -3.7657112 -0.5142089 -0.1465837  0.2520874 12.9474214
##
## Number of Observations: 5579
## Number of Groups: 24
```

```
intervals(cs_mlm)
```

```
## Approximate 95% confidence intervals
##
##  Fixed effects:
##                         lower          est.        upper
## (Intercept)     -4.698744e+00  1.202921e+00 7.104586e+00
## avg_stret        3.196152e-02  3.557731e-02 3.919310e-02
## avg_bikela       1.671579e-02  1.898862e-02 2.126144e-02
## avg_roughn       7.072215e-03  1.249595e-02 1.791969e-02
## citypop         -1.720122e-06 -6.408088e-07 4.385043e-07
## citypopdenssqkm -7.440047e-04  1.346400e-05 7.709327e-04
## AverageTempJul  -3.520788e-01 -8.006031e-02 1.919582e-01
## attr(,"label")
## [1] "Fixed effects:"
##
##  Random Effects:
##   Level: city
##                    lower     est.    upper
## sd((Intercept)) 1.899444 2.616876 3.605287
##
##  Within-group standard error:
##    lower     est.    upper
## 2.594100 2.642804 2.692421
```

Bikescore Components - Fixed Effects Model

```
cs_fe<-lm(pc_bike_2012 ~ avg_stret + avg_bikela + avg_roughn + relevel(city, "New York"), data=bikeScore, na.action=na.exclude)
summary(cs_fe)
```

```
##
## Call:
## lm(formula = pc_bike_2012 ~ avg_stret + avg_bikela + avg_roughn +
##     relevel(city, "New York"), data = bikeScore, na.action = na.exclude)
##
## Residuals:
##     Min      1Q  Median      3Q     Max
## -10.147  -1.355  -0.386   0.671  34.218
##
## Coefficients:
##                                         Estimate Std. Error t value
## (Intercept)                            -4.466819   0.305680 -14.613
## avg_stret                               0.035547   0.001845  19.262
## avg_bikela                              0.018938   0.001160  16.321
## avg_roughn                              0.012332   0.002771   4.451
## relevel(city, "New York")Ann Arbor      3.439057   0.472186   7.283
## relevel(city, "New York")Austin         2.750389   0.225911  12.175
## relevel(city, "New York")Boston         0.614533   0.211653   2.903
## relevel(city, "New York")Calgary        1.475224   0.218943   6.738
## relevel(city, "New York")Chicago        0.671664   0.112872   5.951
## relevel(city, "New York")Eugene        10.251974   0.485781  21.104
## relevel(city, "New York")Fort Collins   7.505239   0.476267  15.758
## relevel(city, "New York")Halifax        3.665711   0.559447   6.552
## relevel(city, "New York")Madison        5.859076   0.376363  15.568
## relevel(city, "New York")Minneapolis    2.967502   0.256312  11.578
## relevel(city, "New York")Moncton        1.500302   0.690075   2.174
## relevel(city, "New York")Montreal       3.652789   0.164290  22.234
## relevel(city, "New York")Portland       5.819035   0.238777  24.370
## relevel(city, "New York")San Francisco  2.064091   0.235079   8.780
## relevel(city, "New York")Saskatoon      2.142025   0.420963   5.088
## relevel(city, "New York")Seattle        3.268538   0.252950  12.922
## relevel(city, "New York")StJohns        1.057221   0.551744   1.916
## relevel(city, "New York")Tempe          3.632179   0.443496   8.190
## relevel(city, "New York")Toronto        1.547705   0.130402  11.869
## relevel(city, "New York")Tuscon         2.444552   0.268462   9.106
## relevel(city, "New York")Vancouver      3.046970   0.259889  11.724
## relevel(city, "New York")Victoria      10.449587   0.643849  16.230
## relevel(city, "New York")Washington     2.058231   0.212198   9.700
##                                        Pr(>|t|)
## (Intercept)                             < 2e-16 ***
## avg_stret                               < 2e-16 ***
## avg_bikela                              < 2e-16 ***
## avg_roughn                             8.73e-06 ***
## relevel(city, "New York")Ann Arbor     3.71e-13 ***
## relevel(city, "New York")Austin         < 2e-16 ***
## relevel(city, "New York")Boston          0.0037 **
## relevel(city, "New York")Calgary       1.77e-11 ***
## relevel(city, "New York")Chicago       2.83e-09 ***
## relevel(city, "New York")Eugene         < 2e-16 ***
## relevel(city, "New York")Fort Collins   < 2e-16 ***
## relevel(city, "New York")Halifax       6.17e-11 ***
## relevel(city, "New York")Madison        < 2e-16 ***
## relevel(city, "New York")Minneapolis    < 2e-16 ***
## relevel(city, "New York")Moncton         0.0297 *
## relevel(city, "New York")Montreal       < 2e-16 ***
## relevel(city, "New York")Portland       < 2e-16 ***
## relevel(city, "New York")San Francisco  < 2e-16 ***
## relevel(city, "New York")Saskatoon     3.73e-07 ***
## relevel(city, "New York")Seattle        < 2e-16 ***
## relevel(city, "New York")StJohns         0.0554 .
## relevel(city, "New York")Tempe         3.22e-16 ***
## relevel(city, "New York")Toronto        < 2e-16 ***
## relevel(city, "New York")Tuscon         < 2e-16 ***
## relevel(city, "New York")Vancouver      < 2e-16 ***
## relevel(city, "New York")Victoria       < 2e-16 ***
## relevel(city, "New York")Washington     < 2e-16 ***
## ---
## Signif. codes:  0 '***' 0.001 '**' 0.01 '*' 0.05 '.' 0.1 ' ' 1
##
## Residual standard error: 2.643 on 5552 degrees of freedom
##   (85 observations deleted due to missingness)
## Multiple R-squared:  0.3667, Adjusted R-squared:  0.3638
## F-statistic: 123.7 on 26 and 5552 DF,  p-value: < 2.2e-16
```

```r
confint(cs_fe)
```

```
##                                                2.5 %      97.5 %
## (Intercept)                             -5.066071488 -3.86756728
## avg_stret                                0.031929283  0.03916483
## avg_bikela                               0.016663192  0.02121253
## avg_roughn                               0.006900242  0.01776425
## relevel(city, "New York")Ann Arbor      2.513387805  4.36472673
## relevel(city, "New York")Austin         2.307514143  3.19326317
## relevel(city, "New York")Boston         0.199610363  1.02945553
## relevel(city, "New York")Calgary        1.046009703  1.90443894
## relevel(city, "New York")Chicago        0.450389756  0.89293829
## relevel(city, "New York")Eugene         9.299653704 11.20429370
## relevel(city, "New York")Fort Collins   6.571568662  8.43890950
## relevel(city, "New York")Halifax        2.568975539  4.76244661
## relevel(city, "New York")Madison        5.121258295  6.59689456
## relevel(city, "New York")Minneapolis    2.465029807  3.46997326
## relevel(city, "New York")Moncton        0.147484437  2.85311915
## relevel(city, "New York")Montreal       3.330715871  3.97486300
## relevel(city, "New York")Portland       5.350937966  6.28713178
## relevel(city, "New York")San Francisco  1.603244096  2.52493839
## relevel(city, "New York")Saskatoon      1.316773246  2.96727699
## relevel(city, "New York")Seattle        2.772657103  3.76441858
## relevel(city, "New York")StJohns       -0.024414278  2.13885583
## relevel(city, "New York")Tempe          2.762753697  4.50160453
## relevel(city, "New York")Toronto        1.292066702  1.80334406
## relevel(city, "New York")Tuscon         1.918261255  2.97084201
## relevel(city, "New York")Vancouver      2.537487228  3.55645366
## relevel(city, "New York")Victoria       9.187390445 11.71178311
## relevel(city, "New York")Washington     1.642239755  2.47422307
```

```r
extractAIC(cs_fe)
```

```
## [1]    27.00 10870.42
```

Figure 2. City Level Analysis

Note. This is not the same data as Table 1. This data is available publicly from BikeScore. It is at the City not the Census Tract level.

Importing Public City Level Data

```r
cityDataPublic <- read.csv("/Users/DogLeg/Dropbox/Data/BikeScore/City_Level_Data_Final.csv")
```

```r
library(ggplot2)

figure1 <- ggplot(cityDataPublic, aes(bikeScore, modeShare)) +
          geom_point(aes(color = factor(country)), size = 3.5) +
          xlab('Bike Score') +
          ylab('Journey to work cycling mode share') +
          stat_smooth(method = "lm", , formula = y ~ x, color="red", se = FALSE) +
          theme(axis.line = element_line(colour = "black"),
          panel.grid.major = element_blank(),
          panel.grid.minor = element_blank(),
          panel.border = element_blank(),
          panel.background = element_blank(),
          axis.title=element_text(size=16,face="bold"),
          axis.text=element_text(size=14)) +
          scale_color_manual(values=c("CA"="#000000", "US"="#919191")) +
          scale_fill_manual(values=c("CA"="#000000", "US"="#919191")) +
        xlim(0, 100)
plot(figure1)
```

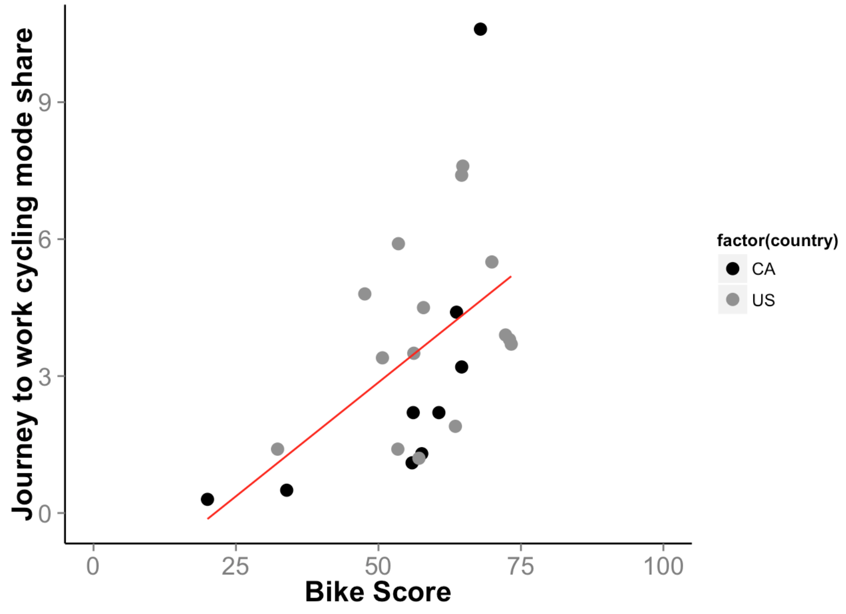


```
ggsave(figure1, file="/Users/DogLeg/Dropbox/Manuscripts/2015 Bikescore/Figure1.pdf", width = 13, height = 10, dpi = 600)
```

Figure 2. Regression models

```
library(ggplot2)

figure2 <- ggplot(bikeScore, aes(x = avg_bikesc, y = pc_bike_2012)) +
          geom_point(fill = "dark grey", colour = "black", alpha = 1/3, size = 2) +
          xlab('Bike Score') +
          ylab('Journey to work cycling mode share') +
          stat_smooth(method=lm,aes(y = fe1fit),colour = "red") +
          theme(axis.line = element_line(colour = "black"),
          panel.grid.major = element_blank(),
          panel.grid.minor = element_blank(),
          panel.border = element_blank(),
          panel.background = element_blank(),
          axis.title=element_text(size=16,face="bold"),
          axis.text=element_text(size=14))
plot(figure2)
```

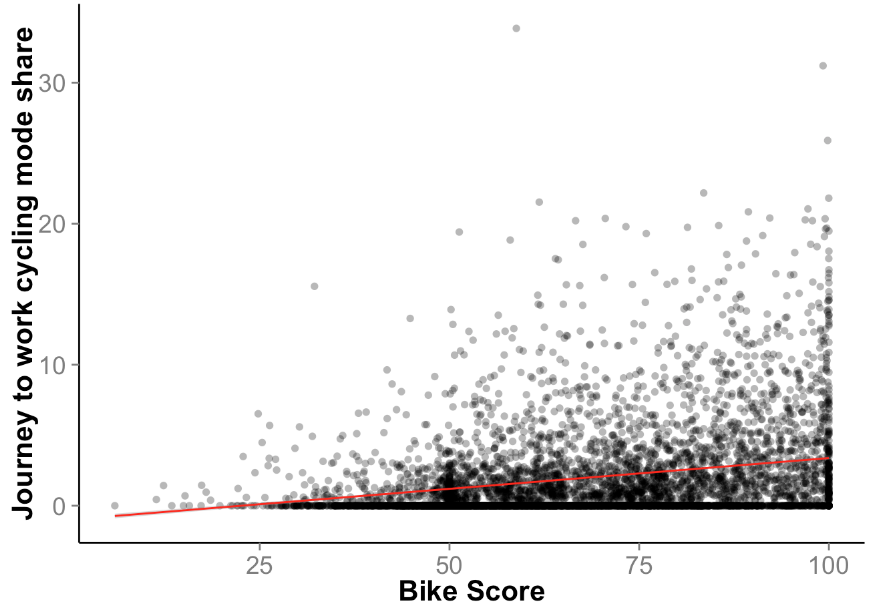


```
ggsave(figure2, file="/Users/DogLeg/Dropbox/Manuscripts/2015 Bikescore/Figure2.pdf", width = 13, height = 10, dpi = 600)
```


Figure 2. Regression models

```
library(ggplot2)
Figure3 <- ggplot(bikeScore, aes(avg_bikesc, pc_bike_2012)) +
          geom_point(fill = "dark grey", colour = "black", alpha = 1/3, size = 2) +
          xlab('Bike Score') +
          ylab('Journey to work cycling mode share') +
          stat_smooth(method = "lm", , formula = y ~ x, color="red", se = FALSE) +
          theme(axis.line = element_line(colour = "black"),
          panel.grid.major = element_blank(),
          panel.grid.minor = element_blank(),
          panel.border = element_blank(),
          panel.background = element_blank(),
          axis.title=element_text(size=16,face="bold"),
          axis.text=element_text(size=14)) +
          facet_wrap (~ city)
plot(Figure3)
```

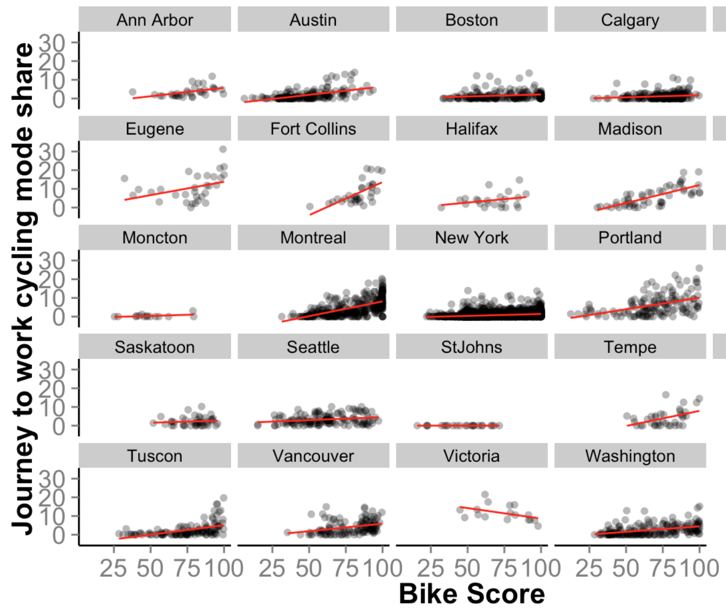


```
ggsave(Figure3, file="/Users/DogLeg/Dropbox/Manuscripts/2015 Bikescore/Figure3.pdf", width = 13, height = 10, dpi = 600)
```

City Specific Regression Estimate

Ann Arbor

```
AnnArbor <- lm(pc_bike_2012 ~ avg_bikesc, subset(bikeScore, city =="Ann Arbor"), na.action=na.exclude)
summary(AnnArbor)
```

```
##
## Call:
## lm(formula = pc_bike_2012 ~ avg_bikesc, data = subset(bikeScore,
##     city == "Ann Arbor"), na.action = na.exclude)
##
## Residuals:
##     Min      1Q  Median      3Q     Max
## -3.2508 -1.2551 -0.3654  0.6037  6.8917
##
## Coefficients:
##             Estimate Std. Error t value Pr(>|t|)
## (Intercept) -3.30650    2.38169  -1.388  0.17494
## avg_bikesc   0.09017    0.03068   2.939  0.00616 **
## ---
## Signif. codes:  0 '***' 0.001 '**' 0.01 '*' 0.05 '.' 0.1 ' ' 1
##
## Residual standard error: 2.407 on 31 degrees of freedom
## Multiple R-squared:  0.218,  Adjusted R-squared:  0.1927
## F-statistic:  8.64 on 1 and 31 DF,  p-value: 0.006162
```

Austin

```r
Austin <- lm(pc_bike_2012 ~ avg_bikesc, subset(bikeScore, city =="Austin"), na.action=na.exclude)
summary(Austin)
```

```
##
## Call:
## lm(formula = pc_bike_2012 ~ avg_bikesc, data = subset(bikeScore,
##     city == "Austin"), na.action = na.exclude)
##
## Residuals:
##     Min      1Q  Median      3Q     Max
## -4.1563 -1.3611 -0.4606  0.6247  9.2712
##
## Coefficients:
##             Estimate Std. Error t value Pr(>|t|)
## (Intercept) -2.45585    0.54650  -4.494 1.33e-05 ***
## avg_bikesc   0.08830    0.01063   8.308 3.81e-14 ***
## ---
## Signif. codes:  0 '***' 0.001 '**' 0.01 '*' 0.05 '.' 0.1 ' ' 1
##
## Residual standard error: 2.349 on 161 degrees of freedom
##   (1 observation deleted due to missingness)
## Multiple R-squared:  0.3001, Adjusted R-squared:  0.2957
## F-statistic: 69.02 on 1 and 161 DF,  p-value: 3.811e-14
```

Boston

```r
Boston <- lm(pc_bike_2012 ~ avg_bikesc, subset(bikeScore, city =="Boston"), na.action=na.exclude)
summary(Boston)
```

```
##
## Call:
## lm(formula = pc_bike_2012 ~ avg_bikesc, data = subset(bikeScore,
##     city == "Boston"), na.action = na.exclude)
##
## Residuals:
##     Min      1Q  Median      3Q     Max
## -2.1361 -1.3548 -0.8473  0.4941 11.6250
##
## Coefficients:
##             Estimate Std. Error t value Pr(>|t|)
## (Intercept) 0.029653   0.715700   0.041    0.967
## avg_bikesc  0.021064   0.009443   2.231    0.027 *
## ---
## Signif. codes:  0 '***' 0.001 '**' 0.01 '*' 0.05 '.' 0.1 ' ' 1
##
## Residual standard error: 2.348 on 170 degrees of freedom
##   (7 observations deleted due to missingness)
## Multiple R-squared:  0.02844,    Adjusted R-squared:  0.02272
## F-statistic: 4.976 on 1 and 170 DF,  p-value: 0.02701
```

Calgary

```r
Calgary <- lm(pc_bike_2012 ~ avg_bikesc, subset(bikeScore, city =="Calgary"), na.action=na.exclude)
summary(Calgary)
```

```
##
## Call:
## lm(formula = pc_bike_2012 ~ avg_bikesc, data = subset(bikeScore,
##     city == "Calgary"), na.action = na.exclude)
##
## Residuals:
##     Min      1Q  Median      3Q     Max
## -1.6374 -1.2171 -0.5548  0.5614  9.7543
##
## Coefficients:
##              Estimate Std. Error t value Pr(>|t|)
## (Intercept) -0.515075   0.709928  -0.726    0.469
## avg_bikesc   0.022831   0.009405   2.427    0.016 *
## ---
## Signif. codes:  0 '***' 0.001 '**' 0.01 '*' 0.05 '.' 0.1 ' ' 1
##
## Residual standard error: 1.812 on 219 degrees of freedom
## Multiple R-squared:  0.0262, Adjusted R-squared:  0.02176
## F-statistic: 5.893 on 1 and 219 DF,  p-value: 0.01601
```

Chicago

```
Chicago <- lm(pc_bike_2012 ~ avg_bikesc, subset(bikeScore, city =="Chicago"), na.action=na.exclude)
summary(Chicago)
```

```
##
## Call:
## lm(formula = pc_bike_2012 ~ avg_bikesc, data = subset(bikeScore,
##     city == "Chicago"), na.action = na.exclude)
##
## Residuals:
##     Min      1Q  Median      3Q     Max
## -2.7198 -0.9972 -0.5826  0.3795 17.7862
##
## Coefficients:
##              Estimate Std. Error t value Pr(>|t|)
## (Intercept) -1.553633   0.319921  -4.856 1.45e-06 ***
## avg_bikesc   0.044956   0.005155   8.721  < 2e-16 ***
## ---
## Signif. codes:  0 '***' 0.001 '**' 0.01 '*' 0.05 '.' 0.1 ' ' 1
##
## Residual standard error: 1.922 on 761 degrees of freedom
##   (5 observations deleted due to missingness)
## Multiple R-squared:  0.09086,    Adjusted R-squared:  0.08967
## F-statistic: 76.06 on 1 and 761 DF,  p-value: < 2.2e-16
```

Eugene

```
Eugene <- lm(pc_bike_2012 ~ avg_bikesc, subset(bikeScore, city =="Eugene"), na.action=na.exclude)
summary(Eugene)
```

```
##
## Call:
## lm(formula = pc_bike_2012 ~ avg_bikesc, data = subset(bikeScore,
##     city == "Eugene"), na.action = na.exclude)
##
## Residuals:
##      Min       1Q   Median       3Q      Max
## -10.5582  -4.9198  -0.3271   3.9518  17.5240
##
## Coefficients:
##             Estimate Std. Error t value Pr(>|t|)
## (Intercept) -0.48951    5.41466   -0.09   0.9286
## avg_bikesc   0.14275    0.06766    2.11   0.0436 *
## ---
## Signif. codes:  0 '***' 0.001 '**' 0.01 '*' 0.05 '.' 0.1 ' ' 1
##
## Residual standard error: 6.829 on 29 degrees of freedom
## Multiple R-squared:  0.1331, Adjusted R-squared:  0.1032
## F-statistic: 4.451 on 1 and 29 DF,  p-value: 0.04362
```

Fort Collins

```
FortCollins <- lm(pc_bike_2012 ~ avg_bikesc, subset(bikeScore, city =="Fort Collins"), na.action=na.exclude)
summary(FortCollins)
```

```
##
## Call:
## lm(formula = pc_bike_2012 ~ avg_bikesc, data = subset(bikeScore,
##     city == "Fort Collins"), na.action = na.exclude)
##
## Residuals:
##     Min      1Q  Median      3Q     Max
## -8.2564 -3.0940 -0.2948  1.7325 10.9696
##
## Coefficients:
##              Estimate Std. Error t value Pr(>|t|)
## (Intercept) -21.76647    6.59665   -3.30  0.00244 **
## avg_bikesc    0.35392    0.07831    4.52 8.47e-05 ***
## ---
## Signif. codes:  0 '***' 0.001 '**' 0.01 '*' 0.05 '.' 0.1 ' ' 1
##
## Residual standard error: 4.717 on 31 degrees of freedom
## Multiple R-squared:  0.3972, Adjusted R-squared:  0.3778
## F-statistic: 20.43 on 1 and 31 DF,  p-value: 8.468e-05
```

Halifax

```
Halifax <- lm(pc_bike_2012 ~ avg_bikesc, subset(bikeScore, city =="Halifax"), na.action=na.exclude)
summary(Halifax)
```

```
##
## Call:
## lm(formula = pc_bike_2012 ~ avg_bikesc, data = subset(bikeScore,
##     city == "Halifax"), na.action = na.exclude)
##
## Residuals:
##     Min      1Q  Median      3Q     Max
## -5.1829 -2.4632 -0.1661  1.5339  9.4947
##
## Coefficients:
##             Estimate Std. Error t value Pr(>|t|)
## (Intercept) -1.04048    3.73857  -0.278    0.783
## avg_bikesc   0.07383    0.05449   1.355    0.190
##
## Residual standard error: 3.793 on 21 degrees of freedom
##   (2 observations deleted due to missingness)
## Multiple R-squared:  0.08041,    Adjusted R-squared:  0.03662
## F-statistic: 1.836 on 1 and 21 DF,  p-value: 0.1898
```

Madison

```
Madison <- lm(pc_bike_2012 ~ avg_bikesc, subset(bikeScore, city =="Madison"), na.action=na.exclude)
summary(Madison)
```

```
##
## Call:
## lm(formula = pc_bike_2012 ~ avg_bikesc, data = subset(bikeScore,
##     city == "Madison"), na.action = na.exclude)
##
## Residuals:
##     Min      1Q  Median      3Q     Max
## -6.1828 -2.6790 -0.0012  2.1639  8.8254
##
## Coefficients:
##             Estimate Std. Error t value Pr(>|t|)
## (Intercept)  -7.2080     1.7245  -4.180 0.000117 ***
## avg_bikesc    0.1924     0.0244   7.886 2.49e-10 ***
## ---
## Signif. codes:  0 '***' 0.001 '**' 0.01 '*' 0.05 '.' 0.1 ' ' 1
##
## Residual standard error: 3.396 on 50 degrees of freedom
##   (1 observation deleted due to missingness)
## Multiple R-squared:  0.5543, Adjusted R-squared:  0.5454
## F-statistic: 62.19 on 1 and 50 DF,  p-value: 2.494e-10
```

Minneapolis

```
Minneapolis <- lm(pc_bike_2012 ~ avg_bikesc, subset(bikeScore, city =="Minneapolis"), na.action=na.exclude)
summary(Minneapolis)
```

```
##
## Call:
## lm(formula = pc_bike_2012 ~ avg_bikesc, data = subset(bikeScore,
##     city == "Minneapolis"), na.action = na.exclude)
##
## Residuals:
##     Min      1Q  Median      3Q     Max
## -4.5734 -2.3147 -0.4526  1.1395 12.1268
##
## Coefficients:
##             Estimate Std. Error t value Pr(>|t|)
## (Intercept)   0.9108     1.6040   0.568   0.5713
## avg_bikesc    0.0383     0.0203   1.887   0.0618 .
## ---
## Signif. codes:  0 '***' 0.001 '**' 0.01 '*' 0.05 '.' 0.1 ' ' 1
##
## Residual standard error: 3.286 on 113 degrees of freedom
## Multiple R-squared:  0.03054,    Adjusted R-squared:  0.02196
## F-statistic:  3.56 on 1 and 113 DF,  p-value: 0.06175
```

Moncton

```
Moncton <- lm(pc_bike_2012 ~ avg_bikesc, subset(bikeScore, city =="Moncton"), na.action=na.exclude)
summary(Moncton)
```

```
##
## Call:
## lm(formula = pc_bike_2012 ~ avg_bikesc, data = subset(bikeScore,
##     city == "Moncton"), na.action = na.exclude)
##
## Residuals:
##     Min      1Q  Median      3Q     Max
## -1.0718 -0.4151 -0.1412  0.1932  2.0102
##
## Coefficients:
##             Estimate Std. Error t value Pr(>|t|)
## (Intercept) -0.80081    0.68519  -1.169   0.2635
## avg_bikesc   0.02361    0.01331   1.774   0.0995 .
## ---
## Signif. codes:  0 '***' 0.001 '**' 0.01 '*' 0.05 '.' 0.1 ' ' 1
##
## Residual standard error: 0.763 on 13 degrees of freedom
## Multiple R-squared:  0.1949, Adjusted R-squared:  0.133
## F-statistic: 3.147 on 1 and 13 DF,  p-value: 0.09948
```

Montreal

```
Montreal <- lm(pc_bike_2012 ~ avg_bikesc, subset(bikeScore, city =="Montreal"), na.action=na.exclude)
summary(Montreal)
```

```
##
## Call:
## lm(formula = pc_bike_2012 ~ avg_bikesc, data = subset(bikeScore,
##     city == "Montreal"), na.action = na.exclude)
##
## Residuals:
##     Min      1Q  Median      3Q     Max
## -8.1455 -2.5429 -0.2881  1.9799 12.2836
##
## Coefficients:
##             Estimate Std. Error t value Pr(>|t|)
## (Intercept) -7.67160    0.95804  -8.008 2.42e-14 ***
## avg_bikesc   0.15817    0.01185  13.349  < 2e-16 ***
## ---
## Signif. codes:  0 '***' 0.001 '**' 0.01 '*' 0.05 '.' 0.1 ' ' 1
##
## Residual standard error: 3.693 on 308 degrees of freedom
##   (10 observations deleted due to missingness)
## Multiple R-squared:  0.3665, Adjusted R-squared:  0.3645
## F-statistic: 178.2 on 1 and 308 DF,  p-value: < 2.2e-16
```

New York

```
NewYork <- lm(pc_bike_2012 ~ avg_bikesc, subset(bikeScore, city =="New York"), na.action=na.exclude)
summary(NewYork)
```

```
##
## Call:
## lm(formula = pc_bike_2012 ~ avg_bikesc, data = subset(bikeScore,
##     city == "New York"), na.action = na.exclude)
##
## Residuals:
##     Min      1Q  Median      3Q     Max
## -1.3954 -0.6548 -0.3816  0.1333 13.5244
##
## Coefficients:
##              Estimate Std. Error t value Pr(>|t|)
## (Intercept) -0.632254   0.110918   -5.70 1.36e-08 ***
## avg_bikesc   0.020277   0.001642   12.35  < 2e-16 ***
## ---
## Signif. codes:  0 '***' 0.001 '**' 0.01 '*' 0.05 '.' 0.1 ' ' 1
##
## Residual standard error: 1.376 on 2116 degrees of freedom
##   (46 observations deleted due to missingness)
## Multiple R-squared:  0.06719,    Adjusted R-squared:  0.06675
## F-statistic: 152.4 on 1 and 2116 DF,  p-value: < 2.2e-16
```

Portland

```
Portland <- lm(pc_bike_2012 ~ avg_bikesc, subset(bikeScore, city =="Portland"), na.action=na.exclude)
summary(Portland)
```

```
##
## Call:
## lm(formula = pc_bike_2012 ~ avg_bikesc, data = subset(bikeScore,
##     city == "Portland"), na.action = na.exclude)
##
## Residuals:
##     Min      1Q  Median      3Q     Max
## -9.6195 -4.1213 -0.5705  2.4191 15.8258
##
## Coefficients:
##             Estimate Std. Error t value Pr(>|t|)
## (Intercept) -2.26290    1.53212  -1.477    0.142
## avg_bikesc   0.12357    0.02116   5.840  3.7e-08 ***
## ---
## Signif. codes:  0 '***' 0.001 '**' 0.01 '*' 0.05 '.' 0.1 ' ' 1
##
## Residual standard error: 5.018 on 135 degrees of freedom
## Multiple R-squared:  0.2017, Adjusted R-squared:  0.1958
## F-statistic: 34.11 on 1 and 135 DF,  p-value: 3.698e-08
```

San Francisco

```
SanFrancisco <- lm(pc_bike_2012 ~ avg_bikesc, subset(bikeScore, city =="San Francisco"), na.action=na.exclude)
summary(SanFrancisco)
```

```
##
## Call:
## lm(formula = pc_bike_2012 ~ avg_bikesc, data = subset(bikeScore,
##     city == "San Francisco"), na.action = na.exclude)
##
## Residuals:
##     Min      1Q  Median      3Q     Max
## -4.4417 -2.3220 -0.6999  1.4773 14.0747
##
## Coefficients:
##             Estimate Std. Error t value Pr(>|t|)
## (Intercept) -3.17442    1.07206  -2.961  0.00345 **
## avg_bikesc   0.07996    0.01342   5.959 1.19e-08 ***
## ---
## Signif. codes:  0 '***' 0.001 '**' 0.01 '*' 0.05 '.' 0.1 ' ' 1
##
## Residual standard error: 3.16 on 193 degrees of freedom
##   (1 observation deleted due to missingness)
## Multiple R-squared:  0.1554, Adjusted R-squared:  0.151
## F-statistic: 35.51 on 1 and 193 DF,  p-value: 1.186e-08
```

Saskatoon

```
Saskatoon <- lm(pc_bike_2012 ~ avg_bikesc, subset(bikeScore, city =="Saskatoon"), na.action=na.exclude)
summary(Saskatoon)
```

```
##
## Call:
## lm(formula = pc_bike_2012 ~ avg_bikesc, data = subset(bikeScore,
##     city == "Saskatoon"), na.action = na.exclude)
##
## Residuals:
##     Min      1Q  Median      3Q     Max
## -2.5566 -1.9257 -0.5252  0.9002  7.8719
##
## Coefficients:
##             Estimate Std. Error t value Pr(>|t|)
## (Intercept)  0.52455    2.77450   0.189    0.851
## avg_bikesc   0.02153    0.03463   0.622    0.538
##
## Residual standard error: 2.387 on 41 degrees of freedom
##   (2 observations deleted due to missingness)
## Multiple R-squared:  0.009335,   Adjusted R-squared:  -0.01483
## F-statistic: 0.3863 on 1 and 41 DF,  p-value: 0.5377
```

Seattle

```
Seattle <- lm(pc_bike_2012 ~ avg_bikesc, subset(bikeScore, city =="Seattle"), na.action=na.exclude)
summary(Seattle)
```

```
##
## Call:
## lm(formula = pc_bike_2012 ~ avg_bikesc, data = subset(bikeScore,
##     city == "Seattle"), na.action = na.exclude)
##
## Residuals:
##     Min      1Q  Median      3Q     Max
## -4.2624 -1.8928 -0.5768  1.7316  6.6781
##
## Coefficients:
##             Estimate Std. Error t value Pr(>|t|)
## (Intercept)  1.35653    0.73312   1.850  0.06653 .
## avg_bikesc   0.03210    0.01147   2.798  0.00592 **
## ---
## Signif. codes:  0 '***' 0.001 '**' 0.01 '*' 0.05 '.' 0.1 ' ' 1
##
## Residual standard error: 2.541 on 130 degrees of freedom
## Multiple R-squared:  0.0568, Adjusted R-squared:  0.04955
## F-statistic: 7.829 on 1 and 130 DF,  p-value: 0.005924
```

Tempe

```
Tempe <- lm(pc_bike_2012 ~ avg_bikesc, subset(bikeScore, city =="Tempe"), na.action=na.exclude)
summary(Tempe)
```

```
##
## Call:
## lm(formula = pc_bike_2012 ~ avg_bikesc, data = subset(bikeScore,
##     city == "Tempe"), na.action = na.exclude)
##
## Residuals:
##     Min      1Q  Median      3Q     Max
## -6.0825 -2.9986 -0.9681  2.1389 12.3238
##
## Coefficients:
##             Estimate Std. Error t value Pr(>|t|)
## (Intercept) -8.29974    4.12788  -2.011  0.05212 .
## avg_bikesc   0.16231    0.05349   3.035  0.00452 **
## ---
## Signif. codes:  0 '***' 0.001 '**' 0.01 '*' 0.05 '.' 0.1 ' ' 1
##
## Residual standard error: 3.976 on 35 degrees of freedom
## Multiple R-squared:  0.2083, Adjusted R-squared:  0.1857
## F-statistic: 9.208 on 1 and 35 DF,  p-value: 0.004523
```

Toronto

```
Toronto <- lm(pc_bike_2012 ~ avg_bikesc, subset(bikeScore, city =="Toronto"), na.action=na.exclude)
summary(Toronto)
```

```
##
## Call:
## lm(formula = pc_bike_2012 ~ avg_bikesc, data = subset(bikeScore,
##     city == "Toronto"), na.action = na.exclude)
##
## Residuals:
##    Min     1Q Median     3Q    Max
## -3.810 -1.953 -1.019  0.221 32.321
##
## Coefficients:
##              Estimate Std. Error t value Pr(>|t|)
## (Intercept) -1.825707   0.673389  -2.711  0.00692 **
## avg_bikesc   0.057035   0.009785   5.829 9.63e-09 ***
## ---
## Signif. codes:  0 '***' 0.001 '**' 0.01 '*' 0.05 '.' 0.1 ' ' 1
##
## Residual standard error: 3.726 on 536 degrees of freedom
##   (6 observations deleted due to missingness)
## Multiple R-squared:  0.05961,    Adjusted R-squared:  0.05786
## F-statistic: 33.98 on 1 and 536 DF,  p-value: 9.635e-09
```

Tuscon

```
Tuscon <- lm(pc_bike_2012 ~ avg_bikesc, subset(bikeScore, city =="Tuscon"), na.action=na.exclude)
summary(Tuscon)
```

```
##
## Call:
## lm(formula = pc_bike_2012 ~ avg_bikesc, data = subset(bikeScore,
##     city == "Tuscon"), na.action = na.exclude)
##
## Residuals:
##     Min      1Q  Median      3Q     Max
## -5.0251 -2.0497 -0.7399  1.2539 14.6203
##
## Coefficients:
##             Estimate Std. Error t value Pr(>|t|)
## (Intercept) -5.04440    1.29214  -3.904 0.000162 ***
## avg_bikesc   0.10144    0.01675   6.055 1.92e-08 ***
## ---
## Signif. codes:  0 '***' 0.001 '**' 0.01 '*' 0.05 '.' 0.1 ' ' 1
##
## Residual standard error: 3.325 on 112 degrees of freedom
##   (1 observation deleted due to missingness)
## Multiple R-squared:  0.2466, Adjusted R-squared:  0.2399
## F-statistic: 36.66 on 1 and 112 DF,  p-value: 1.918e-08
```

Vancouver

```
Vancouver <- lm(pc_bike_2012 ~ avg_bikesc, subset(bikeScore, city =="Vancouver"), na.action=na.exclude)
summary(Vancouver)
```

```
##
## Call:
## lm(formula = pc_bike_2012 ~ avg_bikesc, data = subset(bikeScore,
##     city == "Vancouver"), na.action = na.exclude)
##
## Residuals:
##     Min      1Q  Median      3Q     Max
## -4.6350 -2.7399 -0.8256  1.8551 12.0888
##
## Coefficients:
##             Estimate Std. Error t value Pr(>|t|)
## (Intercept) -2.07104    1.78698  -1.159 0.248917
## avg_bikesc   0.07969    0.02252   3.538 0.000585 ***
## ---
## Signif. codes:  0 '***' 0.001 '**' 0.01 '*' 0.05 '.' 0.1 ' ' 1
##
## Residual standard error: 3.558 on 113 degrees of freedom
## Multiple R-squared:  0.09975,    Adjusted R-squared:  0.09178
## F-statistic: 12.52 on 1 and 113 DF,  p-value: 0.0005854
```

Victoria

```
Victoria <- lm(pc_bike_2012 ~ avg_bikesc, subset(bikeScore, city =="Victoria"), na.action=na.exclude)
summary(Victoria)
```

```
##
## Call:
## lm(formula = pc_bike_2012 ~ avg_bikesc, data = subset(bikeScore,
##     city == "Victoria"), na.action = na.exclude)
##
## Residuals:
##     Min      1Q  Median      3Q     Max
## -5.3058 -2.7488 -1.4566  0.9732  8.6097
##
## Coefficients:
##             Estimate Std. Error t value Pr(>|t|)
## (Intercept) 19.86003    4.37947   4.535 0.000395 ***
## avg_bikesc  -0.11224    0.05754  -1.951 0.070036 .
## ---
## Signif. codes:  0 '***' 0.001 '**' 0.01 '*' 0.05 '.' 0.1 ' ' 1
##
## Residual standard error: 3.945 on 15 degrees of freedom
## Multiple R-squared:  0.2023, Adjusted R-squared:  0.1492
## F-statistic: 3.805 on 1 and 15 DF,  p-value: 0.07004
```

Washington

```
Washington <- lm(pc_bike_2012 ~ avg_bikesc, subset(bikeScore, city =="Washington"), na.action=na.exclude)
summary(Washington)
```

```
##
## Call:
## lm(formula = pc_bike_2012 ~ avg_bikesc, data = subset(bikeScore,
##     city == "Washington"), na.action = na.exclude)
##
## Residuals:
##     Min      1Q  Median      3Q     Max
## -4.0383 -1.7877 -0.7671  1.4215 11.2883
##
## Coefficients:
##              Estimate Std. Error t value Pr(>|t|)
## (Intercept) -1.374991   0.681467  -2.018   0.0451 *
## avg_bikesc   0.058408   0.009774   5.976 1.24e-08 ***
## ---
## Signif. codes:  0 '***' 0.001 '**' 0.01 '*' 0.05 '.' 0.1 ' ' 1
##
## Residual standard error: 2.728 on 176 degrees of freedom
##   (1 observation deleted due to missingness)
## Multiple R-squared:  0.1687, Adjusted R-squared:  0.164
## F-statistic: 35.71 on 1 and 176 DF,  p-value: 1.24e-08
```
