# Supplementary material for: Bike Score®: Associations between urban bikeability and cycling behavior in 24 cities
Source: Int J Behav Nutr Phys Act. 2016 Feb 11;13:18. doi: 10.1186/s12966-016-0339-0 (PMC4751700; doi:10.1186/s12966-016-0339-0)
Supplement: Additional file 2: Table S1. — Characteristics of 24 study cities (city-level variables). (DOCX 15 kb) [file 12966_2016_339_MOESM2_ESM.docx]

**Supplemental Table 1.** Characteristics of 24 study cities (city-level variables)

| City | Country | Journey to work bicycle mode share^a^ | City-wide Bike Score | Population | Population Density  (per km2) | Number of Census Tracts | Average Temp January (°C)^b^ | | Average Temp July  (°C)^b^ | | Total Wet Days (annual)^b^ | | | | | |
| --- | --- | --- | --- | --- | --- | --- | --- | --- | --- | --- | --- | --- | --- | --- | --- | --- |
| Toronto | CA | 2.20% | 56.1 | 2615060 | 4149.5 | 544 | -3.5 | 21.9 | | | | 127.7 | | | | |
| Montreal | CA | 3.20% | 64.6 | 1649519 | 4517.6 | 320 | -9.9 | 20.3 | | | | 124.2 | | | | |
| Calgary | CA | 1.30% | 57.6 | 1096833 | 1329 | 221 | -9.9 | 16.0 | | | | 67.7 | | | | |
| Vancouver | CA | 4.40% | 63.7 | 603502 | 5249.1 | 115 | 2.5 | 17.6 | | | | 153.5 | | | | |
| Saskatoon | CA | 2.20% | 60.6 | 222189 | 1060.3 | 45 | -17.5 | 17.6 | | | | 80.7 | | | | |
| St. Johns | CA | 0.30% | 20 | 106172 | 238 | 26 | -7.8 | 17.6 | | | | 161.4 | | | | |
| Halifax^c^ | CA | 1.10% | 55.9 | 297943 | 1106.5 | 25 | -5.1 | 16.5 | | | | 132.7 | | | | |
| Victoria | CA | 10.60% | 67.9 | 80017 | 4109.4 | 17 | 3.6 | 16.5 | | | | 148.6 | | | | |
| Moncton | CA | 0.50% | 33.9 | 69074 | 489.3 | 15 | -7.2 | 18.7 | | | | 147.3 | | | | |
| New York | US | 1.20% | 57.1 | 8175136 | 10429.5 | 2164 | 0.3 | 23.5 | | | | 121.4 | | | | |
| Chicago | US | 1.40% | 53.4 | 2695598 | 4572.1 | 768 | -4.5 | 23.5 | | | | 129.2 | | | | |
| San Francisco | US | 3.80% | 73 | 805235 | 6632.9 | 196 | 10.6 | 17.1 | | | | 69.3 | | | | |
| Washington, DC | US | 4.50% | 57.9 | 601723 | 3805.6 | 179 | 1.9 | 26.2 | | | | 119.9 | | | | |
| Boston | US | 1.90% | 63.5 | 617594 | 4939.3 | 179 | -1.8 | 22.5 | | | | 109.5 | | | | |
| Austin | US | 1.40% | 32.3 | 790637 | 1024.4 | 164 | 10.6 | 29.5 | | | | 89.5 | | | | |
| Portland | US | 5.90% | 53.5 | 583778 | 1689.3 | 137 | 2.5 | 18.1 | | | | 134.7 | | | | |
| Seattle | US | 3.50% | 56.2 | 608660 | 2799.6 | 132 | 5.2 | 19.2 | | | | 135.5 | | | | |
| Tucson | US | 3.40% | 50.7 | 520116 | 885.8 | 115 | 9.5 | 28.4 | | | | 52.5 | | | | |
| Minneapolis | US | 3.70% | 73.3 | 382578 | 2736.8 | 115 | -9.9 | 21.9 | | | | 118.0 | | | | |
| Madison | US | 4.80% | 47.6 | 233337 | 1172.6 | 53 | -7.8 | 21.9 | | | | 124.2 | | | | |
| Tempe | US | 3.90% | 72.3 | 161746 | 1563.8 | 37 | 11.7 | 32.2 | | | | 39.2 | | | | |
| Ann Arbor | US | 5.50% | 69.9 | 113939 | 1580.7 | 33 | -4.0 | 22.5 | | | | 130.0 | | | | |
| Fort Collins | US | 7.40% | 64.6 | 144000 | 1024.3 | 33 | -0.8 | 22.5 | | | | 88.4 | | | | |
| Eugene | US | 7.60% | 64.8 | 156342 | 1379.2 | 31 | 3.0 | 19.2 | | | | 123.8 | | | | |
| ^a^Canada: 2011 community profiles (for CSD where possible: http://www12.statcan.gc.ca/census-recensement/2011/) | | | | | | | | | |  | | |  | |  | |
| US: Quick Facts, http://quickfacts.census.gov/qfd/states/17/1714000.html | | | | | | | | | |  | | | |  | |  |
| ^b^Climate Research Unit of the Intergovernmental Panel on Climate Change(Intergovernmental Panel on Climate Change 2013), for the most recent time period (1991-2000) | | | | | | | | | |  | | | |  | |  |
| ^c^for Halifax this is 'population centre' area which is the urban zone, as the Census Subdivision is the entire regional municipality for Halifax | | | | | | | | | |  | | | |  | |  |
