# Supplementary material for: Bike Score®: Associations between urban bikeability and cycling behavior in 24 cities
Source: Int J Behav Nutr Phys Act. 2016 Feb 11;13:18. doi: 10.1186/s12966-016-0339-0 (PMC4751700; doi:10.1186/s12966-016-0339-0)
Supplement: Additional file 3: Table S2. — Results of linear multilevel models. (DOCX 13 kb) [file 12966_2016_339_MOESM3_ESM.docx]

**Supplemental Table 2.** Results of linear multilevel models

|  | Null models | | Random Intercept | | Random Slope | |
| --- | --- | --- | --- | --- | --- | --- |
|  | Model A | Model B | Model C | Model D | Model E | Model F |
| **Random Effects** |  |  |  |  |  |  |
| City (std dev, (residual)) | 2.90 (2.82) | 2.90 (2.82) | 2.67 (2.68) | 2.60 (2.68) | 5.42 (2.57) | 5.25 (2.57) |
| Country (std dev) |  | 0.002 |  | <0.001 |  | <0.001 |
| **Fixed Effect** |  |  |  |  |  |  |
| Bike Score (per 10 unit change) |  |  | 0.51 (0.02) | 0.51 (0.02) | 0.78 (0.02) | 0.78 (0.02) |
| AIC | 27523.01 | 27525.01 | 26968.59 | 26960.71 | 26564.46 | 26564.51 |
| logLIK | -13758.51 | -13758.51 | -13480.29 | -13475.36 | -13276.23 | -13273.26 |
| Likelihood Ratio Test | Model B versus A | 0.998 | Model E versus C | <.0001 | Model F versus D | <.0001 |
